# Phosphoproteomic Analysis Reveals the Importance of Kinase Regulation During Orbivirus Infection*[Fn FN2]

**DOI:** 10.1074/mcp.M117.067355

**Published:** 2017-08-29

**Authors:** Bjorn-Patrick Mohl, Edward Emmott, Polly Roy

**Affiliations:** From the ‡Department of Pathogen Molecular Biology, Faculty of Infectious and Tropical Diseases, London School of Hygiene and Tropical Medicine, Keppel Street, London, WC1E 7HT, UK;; §University of Cambridge, Division of Virology, Department of Pathology, Lab block level 5, Box 237, Addenbrookes Hospital, Cambridge, UK

## Abstract

Bluetongue virus (BTV) causes infections in wild and domesticated ruminants with high morbidity and mortality and is responsible for significant economic losses in both developing and developed countries. BTV serves as a model for the study of other members of the *Orbivirus* genus. Previously, the importance of casein kinase 2 for BTV replication was demonstrated. To identify intracellular signaling pathways and novel host-cell kinases involved during BTV infection, the phosphoproteome of BTV infected cells was analyzed. Over 1000 phosphosites were identified using mass spectrometry, which were then used to determine the corresponding kinases involved during BTV infection. This analysis yielded protein kinase A (PKA) as a novel kinase activated during BTV infection. Subsequently, the importance of PKA for BTV infection was validated using a PKA inhibitor and activator. Our data confirmed that PKA was essential for efficient viral growth. Further, we showed that PKA is also required for infection of equid cells by African horse sickness virus, another member of the *Orbivirus* genus. Thus, despite their preference in specific host species, orbiviruses may utilize the same host signaling pathways during their replication.

Reversible protein phosphorylation mediated in part by kinases is a ubiquitous mechanism within cells that facilitates the continual adjustment and tuning of catabolic, anabolic and signal transduction events to maintain cellular equilibrium (1). These attributes make kinases an essential component of host cells to be harnessed for successful viral infection, serving as a mechanism for the regulation of virus entry, transcription, replication, viral RNA binding activity, virus assembly and egress. Examples include Lassa virus (2), Ebola virus (3, 4), Junin virus (5), Andes virus (6), human immunodeficiency virus type 1 (HIV-1) (7), and hepatitis C virus (HCV) (8) where such modulations facilitate successful infection and replication.

Orbiviruses (*Reoviridae* family) are vectored to vertebrate species (*e.g.* sheep, cattle, horses, deer, etc.) by arthropods (gnats, ticks, or mosquitoes depending on the virus) hence determining their geographic distribution. Bluetongue virus (BTV)^1^ with 27 serotypes is one of the most widespread pathogen of ruminants (mortality reaching 70% in sheep) in many parts of the world and acts as an important representative of orbiviruses (9). African Horse Sickness virus (AHSV), which predominantly infects equids with 95% mortality in horses, is genetically and morphologically like BTV. Replication of these two viruses in such distinct cell types provides an opportunity to dissect the critical virus-host interactions that occur in each. BTV (and AHSV) is a nonenveloped, icosahedral double-capsid virus with an architecturally complex structure. Surrounding a genome of 10 segmented double-stranded RNA (dsRNA) genome, are two concentric protein shells composed of 7 structural proteins (VP1-VP7) (10). Additionally, 4 nonstructural proteins (NS1-NS4) are also synthesized in the infected host cells and each plays important roles in the virus life cycle (11, 12).

Recent studies in our lab have highlighted the significance of kinases for the BTV life-cycle. Casein kinase 2 (CK2) was shown to mediate the phosphorylation of the viral protein NS2. Inhibition of CK2 activity, but not CK1 activity, was shown to be deleterious to virus replication (13, 14). CK2 has also been reported to be involved for phosphorylation of one of the non-structural proteins, NSP5, of Rotavirus, a *Reoviridae* family member, whereas CK1α was essential for NSP5 hyperphosphorylation (15, 16). Given the limited information available to date and based on this example of a host kinase facilitating BTV infection, we interrogated the phosphoproteome of BTV infected HeLa cells to identify intracellular signaling pathways and critical host factors activated or suppressed upon BTV infection. A series of kinases were identified as a result of BTV infection. We chose one of these kinases, protein kinase A (PKA), which constituted a novel host factor that had not been previously associated with BTV. To determine whether PKA activity held similar significance to BTV, as had been previously documented for HCV (17), adenovirus (18), and Herpes simplex virus 1 (19).

Functional studies using a PKA inhibitor showed impaired viral replication in both HeLa and sheep PT cells, a natural host derived cell line. Furthermore, PKA inhibition also served to impair AHSV replication in HeLa and equid dermal cells, a natural host derived cell line. Conversely, PKA activation had the opposite effect on both BTV and AHSV. This similarity in response may suggest that both BTV and AHSV share a dependence on these PKA regulated pathways. Further, we also examined AKT/protein kinase B (PKB) activity, which featured in our phosphoproteome analysis and that had recently been implicated in mediating autophagy induction by BTV (20). Our data showed an increase in AKT substrate phosphorylation during both BTV and AHSV infection, which in the case of BTV diminished during the infection.

## EXPERIMENTAL PROCEDURES

### 

#### 

##### Cell lines and viruses

BSR cells (BHK-21 subclone) (ATCC® CCL10™), HeLa cells (HeLa, ATCC® CCL-2™) and sheep PT cells (ovine-derived kidney cells, ATCC® Number: CRL-1633™) were maintained in Dulbecco's modified Eagle's medium (DMEM) (Sigma-Aldrich) Equine dermal (E. Derm) cells (NBL-6, ATCC CCL-57) were cultured in Eagle minimum essential medium (MEM; Sigma). Both media were supplemented with 10% (v/v) fetal bovine serum (FBS; Invitrogen), 100 U of penicillin/ml, and 100 μg of streptomycin/ml (Sigma-Aldrich) and MEM Non-Essential Amino Acids (Gibco). BTV serotype 1 (BTV-1) and AHSV serotype 1 (AHSV-1) stock was obtained by infecting BSR cells at a low multiplicity of infection (MOI) and harvested when a 100% cytopathic effect was evident. Virus stocks were stored at 4 °C.

##### Pharmacological Reagents

H-89 (PKA inhibitor) (tlrl-h89, Invivogen), Dibutyryl-cAMP (PKA activator) (sc-201567, Santa Cruz Biotechnology, Inc), Akt Inhibitor VIII (AKT inhibitor) (124018–1MG, Merck). All reagents were used at the concentrations specified.

##### Infection Techniques

For studies of viral infection, HeLa, PT and E. derm cell monolayers were washed with FBS-free medium and infected with BTV or AHSV at an MOI of 5. Virus adsorptions were carried out for 30 min at 4 °C, followed by incubation at 37 °C in growth medium (in the presence or absence of pharmacological activator or inhibitors) for 12, 18, 24, and 36 h.

##### Plaque Assays

BSR cells were grown in 6 well plates and infected with virus recovered from previously infected cells that had been treated with a carrier medium, activator or inhibitors, as described above. Following adsorption for 30 min at 4 °C, cells were incubated at 37 °C in growth medium for 1 h. Growth medium was removed and replaced by 0.6% Avicel (FMC BioPolymer) overlay medium (Eagle's MEM containing l-glutamine, 10% FBS and antibiotics). Cells were incubated at 37 °C for 72 h before being fixed with 4% paraformaldehyde and subsequently stained with crystal violet. Titers were expressed as plaque forming units per ml (Pfu/ml).

##### Western Blot Analysis

SDS-PAGE gels were transferred via a semi-dry blotter to PVDF transfer membranes and blocked for 4 h with TBS-T containing 10% (w/v) milk powder. Primary antibodies that were used for the detection of BTV included NS2 (Guinea pig anti-NS2 serum), VP5 (Guinea pig anti-VP5 serum), NS1 (Rabbit anti-NS1) and GAPDH (rabbit anti-GAPDH (ab9485; Abcam) as a host cell marker. AHSV NS2 protein was detected using a polyvalent serum (horse-derived). Further, rabbit anti-Phospho-PKA substrate (RRXS/T) (100G7E) (9624, Cell Signaling) and rabbit anti-Phospho-AKT substrate RXRXXS/T) (110B7E) (9614, Cell Signaling) were used to detect phosphorylated substrates. Primary antibodies were added to membranes and incubated overnight at 4 °C. Secondary antibodies (IgG, 1:10,000) were alkaline phosphatase-conjugated goat anti-guinea pig immunoglobulin G (Sigma-Aldrich, A5062), goat anti-rabbit (Sigma-Aldrich, A0418) and rabbit-anti-horse (Sigma-Aldrich, A6063).

##### SILAC Experiment

HeLa cells were SILAC labeled using SILAC medium (R0K0, R6K4, R10K8) from Dundee Cell Products for eight cell passages. Cells were infected with an MOI of 5. At 12 h and 18 h post infection (h.p.i), cells were washed with PBS and lysed using lysis buffer (8 m Urea, 75 mm NaCl, 50 mm Tris, pH 8.2, Protease inhibitor mixture (Promega), 1 mm NaF, 1 mm Beta-glycerophosphate, 1 mm orthovanadate, 10 mm sodium pyrophosphate and 1 mm PMSF). Cellular debris was spun down and lysates combined in a 1:1:1 ratio prior to and subjected to phosphopeptide enrichment and analysis via mass spectrometry (University of Bristol).

##### LC-MS/MS and Sample Preparation

SILAC pools were reduced (20 mm TCEP for 1 h at 55 °C), alkylated (35 mm iodoacetamide for 30min at room temperature) and proteins precipitated using 6 volumes of acetone (15 h, −20 °C). Precipitated proteins were resuspended in 100 mm TEAB to which 2.5% (w/w) trypsin was added and the samples incubated overnight at 37 °C. Following digestion, the samples were resuspended in 5% formic acid and desalted using SepPak cartridges according to the manufacturer's instructions (Waters, Milford, MA). 1 mg of Eluate from the SepPak cartridge was first desiccated and then resuspended for either TiO_2_-based or Fe-NTA-based phosphopeptide enrichment (0.5 mg was used for each) according to the manufacturer's instructions (Pierce). Enriched phosphopeptides were then fractionated using a Dionex Ultimate 3000 nanoHPLC system in line with an LTQ-Orbitrap Velos mass spectrometer (Thermo Scientific). In brief, peptides in 1% (v/v) formic acid were injected onto an Acclaim PepMap C18 nano-trap column (Thermo Scientific). After washing with 0.5% (v/v) acetonitrile 0.1% (v/v) formic acid peptides were resolved on a 250 mm × 75 μm Acclaim PepMap C18 reverse phase analytical column (Thermo Scientific) over a 150 min organic gradient, using 7 gradient segments (1–6% solvent B over 1min., 6–15% B over 58min., 15–32%B over 58min., 32–40%B over 5min., 40–90%B over 1min., held at 90%B for 6min and then reduced to 1%B over 1min, with a flow rate of 300 nl/min. Solvent A was 0.1% formic acid and Solvent B was aqueous 80% acetonitrile in 0.1% formic acid. Peptides were ionized by nano-electrospray ionization at 2.1 kV using an emitter with an internal diameter of 30 μm (Thermo Scientific) and a capillary temperature of 250 °C. Tandem mass spectra were acquired using an LTQ- Orbitrap Velos mass spectrometer controlled by Xcalibur 2.1 software (Thermo Scientific) and operated in data-dependent acquisition mode. The Orbitrap was set to analyze the survey scans at 60,000 resolution (at *m*/*z* 400) in the mass range *m*/*z* 300 to 1800 and the top 12 multiply charged ions in each duty cycle selected for MS/MS in the LTQ linear ion trap. Charge state filtering, where unassigned precursor ions were not selected for fragmentation, and dynamic exclusion (repeat count, 1; repeat duration, 30 s; exclusion list size, 500) were used. Fragmentation conditions in the LTQ were as follows: normalized collision energy, 35%; activation q, 0.25; activation time 10ms; and minimum ion selection intensity, 500 counts. Multistage activation was enabled with neutral loss masses of 32.7, 49, and 98.

##### Data Analysis

The raw data files were processed and quantified using Maxquant v1.5.7.4 (21) and searched against the Uniprot Human database (70,550 entries, downloaded September 19^th^, 2016) plus a custom fasta file generated in-house containing the BTV protein sequences (South African reference strain, Genbank accession numbers FJ969719-FJ969728) which is included as part of PRIDE submission PXD005550. Precursor mass tolerance was set at 4.5ppm, and MS/MS tolerance was set at 0.5Da. Search criteria included carbaminomethylation of cysteine as a fixed modification. Oxidation of methionine, N-terminal acetylation, and STY phosphorylation were selected as variable modifications. Quantification was based on Light (Arg 0, Lys 0), Medium (Arg 6, Lys 4), and Heavy (Arg 10, Lys 8) SILAC labels. Searches were performed with full tryptic digestion, a minimum peptide length of 7 amino acids, and a maximum of 2 missed cleavages were allowed. The reverse database search option was enabled and the maximum false discovery rate for both peptide and protein identifications was set to 0.01. Quantitation was performed using a mass precision of 2 ppm and the requantify option in Maxquant was enabled. The full Maxquant output is provided as part of PRIDE submission PXD005550 permitting viewing of annotated spectra in Maxquant v1.5.7.4. Downstream analysis was accomplished in the Perseus software (22). In all cases, contaminants and reverse database hits were removed. Downstream phosphosite analysis was restricted to those with a localization probability of >0.75 and a score difference of over 5 (also referred to as class I phosphosites). Protein, peptides or site ratios were converted to log_2_ and the mean log_2_ ratios across the three replicates were calculated. Only data obtained for at least two of the three biological replicates were used for further analysis, this would exclude a peptide identified in both the TiO_2_ and Fe-NTA samples from an individual biological replicate.

Weblogo (23) was used to produce sequence logos looking at phosphorylation site motifs. To identify significantly altered phosphorylation patterns, phosphorylation sites (supplemental Table S3) which showed a significant alteration in the abundance by *t* test (*p* < 0.05) were analyzed using the Motif-X software (24). The settings used were width 13, Occ 20, and central S/T. Phosphotyrosine enrichment was not investigated using motif-x because of the low numbers of identified phosphosites. Motif-X analysis is shown in supplemental Table S6.

Gene ontology analysis was performed using STRING version 10.5 (https://string-db.org) (25) using phosphoproteins identified as regulated based on their inclusion of at least one regulated phosphosite. These data are listed in supplemental Table S5.

Phoxtrack (26) was used to test for enrichment of activated or inhibited kinases in the sample. Phosphosite windows (± 6 amino acids) and mean log_2_ ratios were uploaded to the software. Phoxtrack was set to require a minimum of two phosphosites per kinase, and 10,000 permutations. The human PhosphoSitePlus, SWISS-Prot, HPRD and Phospho.ELM were selected as the databases for screening. Phoxtrack kinase and substrate analysis are shown in supplemental Table S7.

##### Experimental Design and Statistical Rationale

The phosphoproteomic experiment was performed with 3 independent biological replicates, with each replicate divided into two aliquots for parallel enrichment of phosphopeptides using either TiO_2_ or FeNTA. These were performed using SILAC-labeled cells, allowing comparison of mock infected cells (12h) with BTV infected cells at either 12 h.p.i or 18 h.p.i. Label-switching was performed in one replicate (replicate 2) to control for any impact of the SILAC labeling on the cell culture. Mass spectrometry was performed on infected human HeLa cells, rather than the more usual ovine host cells, because of superior annotation and analysis support for human proteomic data. For stringency, downstream analysis was restricted to high-confidence class I phosphosites, and both phosphoproteins and phosphosites had to be identified in at least 2/3 experiments to be used for downstream analysis. Analysis was performed as described in data analysis.

##### Quantification of Protein Levels and Phosphorylation by Densitometry

Immunoblots were scanned and analyzed via semi-quantified densitometry, using ImageJ, available through the National Institutes of Health.

## RESULTS

### 

#### 

##### Phosphoproteomic Analysis of BTV Infected HeLa Cells Reveals Changes in Cellular Protein Phosphorylation Profiles

**Fig. 1.**
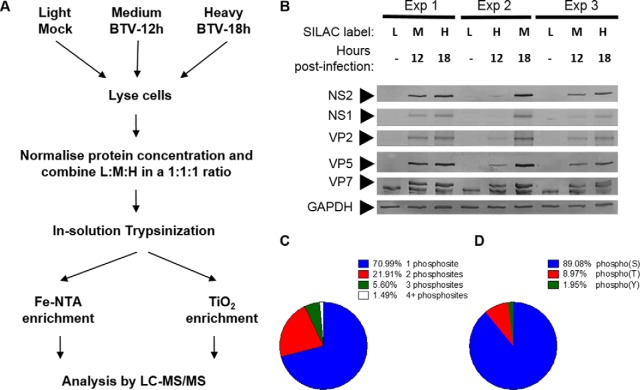
**Experimental design and validation of infection with BTV.** *A*, A schematic representation of the phosphoproteomic experimental design is shown. The use of medium and heavy media for the 12h and 18h samples respectively was observed for experiments 1 and 3, and in experiment 2 the media was switched to control for potential impacts on cell growth or infection. *B*, Infection conditions within the various samples submitted for LC-MS/MS analysis were confirmed by Western blotting against viral antigens indicated on the left. Cellular GAPDH was used as a loading control. *C*, The number (percentage of the table) and *D*, percentage identity of phosphorylation sites within the total phosphoproteomic dataset obtained is shown.

HeLa cells were labeled with light (R0K0), medium (R6K4), and heavy (R10K8) amino acids. Cells were mock infected (R0K0) and infected (R6K4 and R10K8) with BTV (MOI = 5) and harvested at either 12 h.p.i or 18 h.p.i. Successfully infected cell lysates within each replicate experiment were combined, trypsin digested and enriched for phosphopeptides, followed by LC-MS/MS (Fig. 1*A*). Lysates from three replicate experiments were analyzed on SDS-PAGE gels and Western blots carried out to confirm the successful infection of cells. HeLa cells infected with BTV (R6K4 and R10K8) demonstrated the presence of viral proteins, including the structural proteins VP2, VP5, VP7, and the nonstructural protein NS1 and NS2, confirming successful viral entry and replication. Viral protein concentrations increased concurrently to the temporal progression of the infection between 12 h.p.i and 18 h.p.i (Fig. 1*B*).

Phosphopeptide enrichment was carried out using complementary Fe-NTA and Ti0_2_ enrichment procedures. The phosphoproteins, phosphopeptides, and high confidence phosphosites identified by this analysis are detailed in supplemental Tables S1–S3 respectively. Summary data is presented in Table I. Of the 4823 peptides identified, 1384 phosphopeptides were identified, corresponding to 29% of the total. 704 (50.9%) of these phosphopeptides were common to both enrichment methods (supplemental Fig. S1*B*). Two hundred peptides were unique to the Fe-NTA enrichment, whereas TiO_2_ enrichment identified a further 480 phosphopeptides, which had not been identified following Fe-NTA enrichment. The identified peptides corresponded to 1599 proteins, of which 598 represented phosphoproteins (supplemental Table S1). Experimental replicates showed high correlation between replicates (supplemental Fig. S2) with Pearsons correlation coefficients in the range 0.786–0.835. No significant difference in enrichment of single or multiple phosphorylated peptides was observed between the two enrichment methods (supplemental Fig. S1*C* and S1*D*). Overall, 70% of the phosphopeptides had a single phosphorylated residue, 25% had two, and the remainder three or four (Fig. 1*C*). The phosphosites could be divided into 89% Ser, 9% Thr, and 2% Tyr phosphorylation sites (Fig. 1*D*) which is comparable with other global phosphoproteomic studies (27). Phosphosites were further processed to ensure only those with a high localization probability and those identified in multiple biological repeats were used for downstream kinase analysis. These 922 high-confidence phosphosites are detailed in supplemental Table S3. Although a majority of viral proteins were detected confirming Western blotting data (Fig. 1*B*) of a successful infection, no phosphorylated viral peptides were observed in this analysis. As expected, only a small subset of phosphosites show differential regulation upon BTV infection (supplemental Fig. S3), with the majority showing negligible change. Full details on the relative levels of individual phosphosites are shown in supplemental Table S3. supplemental Table S4 details all the phosphoproteins, phosphopeptides, and phosphosites that were considered to be significantly regulated by BTV infection (*t* test, *p* < 0.05).

**Table I.** Summary data A summary of the various numbers of proteins, peptides and phosphosites identified in this manuscript. Numbers exclude contaminants, the proteins and peptides listed are nonredundant across the three experiments, and to be classed as a high confident phosphosite, a site needed to be identified with a localization score of >0.75 and in at least 2 of the 3 experiments.

**Phosphoproteins (598)**	
Phosphoproteins with 1+ regulated phosphosites	48.5% (290)
12h/Mock	34.9% (209)
18h/Mock	36.6% (219)
18h/12h	11.7% (70)
**Phosphopeptides (1384)**	
Regulated phosphopeptides	35.4% (490)
12h/Mock	23.4% (324)
18h/Mock	23.8% (330)
18h/12h	6.7% (93)
**High confidence phosphosites (922)**	
Regulated phosphosites	53.5% (493)
12h/Mock	35.2% (325)
18h/Mock	36.0% (332)
18h/12h	9.9% (91)

Gene ontology (GO) analysis of phosphoproteins significantly regulated by BTV infection was performed using the STRING database (supplemental Table S5). Analysis revealed most phosphoproteins participating in biological processes involving cellular or RNA metabolism. However, cell cycle processes (FDR 6.22E-08) and proteins involved in viral processes (1.76E-07) were also identified. Previous studies on BTV infection revealed cell cycle disregulation so these data are consistent with prior observations (28). GO Molecular functions of regulated phosphoproteins showed an over-representation of RNA or nucleic-acid binding proteins, and analysis of cellular localization (GO Cellular component) suggested nuclear proteins dominated. KEGG analysis identified only a single hit, with spliceosome components overrepresented.

**Fig. 2.**
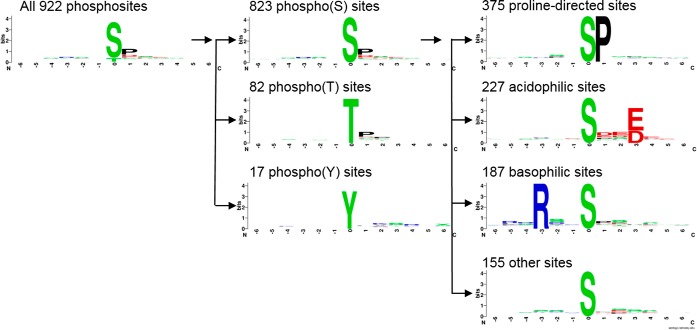
**Identification of sequence motifs within the phosphoproteome of BTV-infected cells.** The 922 high confidence phosphosites (see supplemental Table S3) were analyzed with sequence logos prepared using the weblogo software. These could be further divided into phospho(S/T/Y) sites and the phospho(S) sites divided down further still into proline-directed, acidophilic, basophilic or other sites.

Motif analysis using sequence logos of the 922 phosphorylation sites identified within the dataset is presented in Fig. 2. Phospho-serine sites dominate with 89% of the identified sites, followed by phospho-threonine (9%) and phospho-tyrosine (2%). The phospho-serine sites can be further divided into proline-directed (46%), acidophilic (28%), basophilic (23%) and other sites (19%) though there can be some cross-over between these categories. The proportions observed were consistent with previous studies using this approach (29). Proline-directed kinases include MAPK, CDK, ERK, JNK, and GSK. The acidophilic motif identified is consistent with CK2 substrates or polo-like kinases (30). Basophilic motifs, including an arginine at the −3 position, are targets of a range of kinases including PKA, Akt, and AMPK (31, 32). When investigating proteins showing significant alterations in their abundance (*t* test, *p* < 0.05), proline-directed motifs dominate, which is consistent with their over-representation within the data set (supplemental Fig. S4*A*–S4*C*). However, when the later 18 h time point is compared with the 12 h time point, it is clear that some other motifs are increasing in abundance with the appearance of arginine at the −3 position in the sequence logo suggesting regulation of basophilic motifs (supplemental Fig. S4*C*). Although information can be gained by sequence logos in this manner, they do lack detail on minor variants, requiring more detailed investigation.

To elucidate the kinases involved, we used two complementary approaches, X-track motif analysis to identify significantly over-represented motifs (24) and Phoxtrack (PHOsphosite-X-TRacing Analysis of Causal Kinases) (26). The Phoxtrack software uses non-parametric statistics to determine whether defined kinase specific sets of phosphosites, derived from a selection of public databases, show significant and concordant differences in their abundance between biological samples.

Motif-X was used to identify sequence motifs from the phosphosite data based on a −6 to +6 sequence window around the phosphorylated residue at position 0. T-tests were performed on the high confidence phosphosites identified (supplemental Table S3) and those showing significantly altered abundance (*p* < 0.05) were used for motif analysis (supplemental Table S6). Motif analysis was also performed on the total phosphosite dataset from supplemental Table S3 to identify the full range of motifs enriched within this experiment. No threonine motifs were identified as significantly regulated by the software, most likely because of the low proportion of phosphothreonine peptides in the dataset. Tyrosine motifs were omitted from the analysis for this reason. However, several serine-centered motifs were identified as regulated by BTV at both 12 h.p.i and 18 h.p.i, which corresponded to known phosphorylation motifs. Akt-based motifs (Rxx*S*) showed regulation by BTV, as did CK (*S*DxE), GSK3 (Sxxx*S*P) and ERK1/2 (*S*P). Except for GSK3, these kinases have been shown to have a role in BTV infection previously (14, 33) suggesting the approach has successfully confirmed previous results, as well as identifying a potentially novel kinase.

For a more sensitive approach, we applied Phoxtrack to investigate the high-confidence phosphosites (supplemental Table S3) to identify both kinases and kinase substrates exhibiting significant enrichment (Fig. 2). (supplemental Table S7 shows the complete list of data obtained. Note that as the different databases searched by phoxtrack use different nomenclature, the same kinase can be identified from different databases or under different names. Prospective novel kinases that were identified included p21-activated protein kinase 2 (PAK2), Protein kinase C delta type (PKCD), PAS Domain Containing Serine/Threonine Kinase (PASK), Death-associated protein kinase 1 (DAPK1), Ribosomal Protein S6 Kinase A1 and 3 (RPS6KA1 and 3), Protein Kinase A (PKA) and Polo Like Kinase 3 (PLK3) (Table II).

**Table II.** Kinase regulation during BTV infection The Phoxtrack software was used to identify significantly regulated kinases from the total set of phosphosite motifs identified. The software identifies the same kinase multiple times if this is found in several databases, here only the highest scoring hit is shown. The full data set is given in Table S7. The heading details the kinase identified, the database used for identification, the number of phosphosites used, normalized enrichment value, *p* value, and where a report of kinase regulation by BTV has previously been made, the reference.

Kinase	Database	No. of phosphosites	NEV	*p* value	Reference
**12 h Upregulated**					
PAK2	HPRD	4	2.32	0	
P70S6K	PSP	6	2.3	6.00E-04	(20)
CDK1	PSP	20	1.99	0.005	
PKCD	PSP	4	1.93	0.0046	
PASK	Swiss	2	1.78	0.0022	
DAPK1	Swiss	2	1.77	0.0034	
RPS6KA3	Swiss	2	1.77	0.0034	
RPS6KA1	Swiss	2	1.77	0.002	
CK	Swiss	2	1.58	0.0387	(14)
PRKCA (PKA)	HPRD	2	1.56	0.0489	
**12 h Downregulated**					
TESK2	HPRD	2	−1.84	0	
LIMK1	PSP	2	−1.83	4.00E-04	
LIMK2	PSP	2	−1.83	0	
P38-ALPHA (MAPK14)	PSP	4	−1.76	0.0166	(66, 67)
JNK2	PSP	3	−1.56	0.0471	(67)
**18 h Upregulated**					
P70S6K	PSP	6	2.55	2.00E-04	(20)
CDK1	PSP	20	2.5	0.001	
PAK2	HPRD	4	2.43	0	
RSK_GROUP (RPS6K)	PELM	4	2.18	6.00E-04	
PKCD	PSP	4	1.93	0.0054	
CDK_GROUP	PELM	2	1.84	0	
PASK	Swiss	2	1.77	0.0024	
PKACA (PKA)	PSP	6	1.77	0.0169	
DAPK1	Swiss	2	1.77	0.0036	
**18 h Downregulated**					
PLK3	PSP	3	−2.08	4.00E-04	
JNK2	PSP	3	−2.07	6.00E-04	(67)
MAPK14	Swiss	3	−2.06	4.00E-04	
ERK2	PSP	3	−1.98	0.003	(20)
MAP2K_GROUP	PELM	2	−1.82	0.001	
RET	PSP	2	−1.82	6.00E-04	
MAPK11	Swiss	2	−1.74	0.006	
MAPK8 (JNK1)	PELM	2	−1.73	0.0083	(67)
ERK1	PSP	6	−1.69	0.0273	(20)
**18 h over 12 h Upregulated**					
P90RSK	PSP	5	2.15	0.0014	
PAK2	HPRD	4	1.94	0.0058	
LIMK2	PSP	2	1.84	4.00E-04	
TESK2	HPRD	2	1.83	2.00E-04	
LIMK1	PSP	2	1.82	0	
P70S6K	PSP	6	1.81	0.0138	(20)
CDK1	PSP	20	1.76	0.0208	
PDK1	PSP	3	1.58	0.0455	
PAK1	PELM	2	1.57	0.0455	
**18 h over 12 h Downregulated**					
MAPK14	Swiss	3	−2.15	0	(67)
RET	PSP	2	−1.84	0	
ERK1	PSP	6	−1.84	0.0113	(20)
MAP2K_GROUP	PELM	2	−1.82	0	
MAPK9 (JNK2)	HPRD	2	−1.8	0.001	(67)
MAPK11	Swiss	2	−1.79	6.00E-04	

At both 12 h.p.i and 18 h.p.i, phosphorylated ribosomal protein S6 kinase beta-1 (p70S6 kinase), PKA, Protein Kinase C (PKC) and CDK1/2 substrates were enriched. MAPK14 (P38-MAPK) and JNK2 substrates were reduced in their abundance (Table II). Many of the kinase substrates at 18 h showed an increase on their 12 h phenotype such as p70S6K and CDK1. In other cases, several kinase substrates only showed significant differences at 18 h.p.i such as decreased ERK1/2 and PLK3. Notably several kinases show diminished activity at 12 h.p.i compared with mock, but activity appears restored by 18 h.p.i. LIMK1 and 2, and TESK, show this pattern. One weakness of the phoxtrack approach is that only a single abundance value can be applied. Here we used average fold change from a minimum of 2 of our 3 experiments. However, this does mean that data on variation between experiments is lost and not considered when determining kinase enrichment with this method. Between the phoxtrack and motif-x analyses, most of the previously described impacts of BTV infection on kinase activity have been recapitulated in this dataset, and prospective novel kinases identified.

##### Depletion of PKA Phosphorylated Substrates Following Infection with BTV Reduces Viral Replication, Whereas Enrichment of PKA Phosphorylated Substrates Enhances Viral Replication

Because our phosphoproteomic analysis identified PKA, also known as cAMP-dependent protein kinase, as a novel kinase active during BTV infection, we wanted to validate and assess its role during BTV replication. To this end, we used the PKA inhibitor H89 (34) and the PKA activator Dibutyryl-cAMP (35) to explore the effects of PKA inhibition and activation on BTV replication. Although HeLa cells support BTV replication successfully and were chosen for the initial phosphoproteomic characterization of BTV infection, because of the superior annotation of the human proteome and availability of analysis tools (Fig. 1), it was necessary to complement our analysis by carrying out replicate experiments using a natural host cell line, such as sheep derived PT cells.

To determine the effects of inhibition or activation of PKA activity on BTV protein levels, the lysates of HeLa and sheep PT cells infected with BTV were analyzed. Cells were treated 1 h.p.i with DMSO, 40 μm H89, or 1 mm Dibutyryl-cAMP and harvested at 12 h.p.i and 18 h.p.i. for analysis by Western blotting. Representative Western blots are shown for 12 h.p.i (supplemental Fig. S5*A*) and 18 h.p.i. (supplemental Fig. S5*B*).

**Fig. 3.**
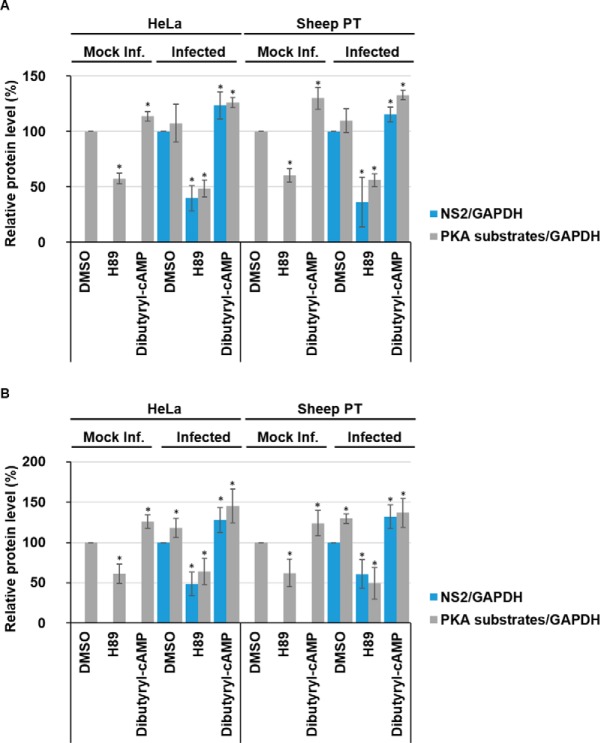
**Inhibition of PKA reduces BTV replication whereas further stimulation of PKA enhances BTV replication.** HeLa and sheep PT cells infected with BTV1 (MOI = 5) were treated 1 h.p.i with 40 μm H89 or 1 mm Dibutyryl-cAMP and harvested 12 h.p.i (*A*) and 18 h.p.i (*B*). Mock infected and DMSO treated cells were included as controls. Samples were analyzed by Western blot and densitometry analysis of phosphorylated-PKA substrates and NS2. Results are expressed as the relative protein levels as indicated on the left. Error bars represent the S.D. values of stimulations from three independent experiments. A star (*) denotes a significant difference from control (*p* < 0.05).

Quantification of Western blots via densitometry for samples collected 12 h.p.i (Fig. 3*A*) validated the effects of H89 and Dibutyryl-cAMP on the levels of phosphorylated PKA substrates normalized to host cell GAPDH levels in mock, or noninfected, HeLa or sheep PT cells. Mock infected cells treated with the H89 inhibitor showed a significant decrease in phosphorylated PKA substrates compared with DMSO-only treated cells. Phosphorylated PKA substrates decreased by ∼43% (± 5%) in HeLa cells and by ∼40% (± 6%) in sheep PT cells (Fig. 3*A*). Concurrently, cells treated with the Dibutyryl-cAMP activator showed a significant increase in phosphorylated PKA substrates compared with DMSO-only treated cells. Phosphorylated PKA substrates increased by ∼14% (± 4%) in HeLa cells and by ∼30% (± 10%) in sheep PT cells (Fig. 3*A*).

At 12 h.p.i there is no significant increase of phosphorylated PKA substrates in infected HeLa or sheep PT cells, when compared with mock infected cells (Fig. 3*A*). This observation was consistent with our phosphoproteome data (supplemental Table S1–S5). However, BTV infected cells treated with the H89 inhibitor showed significant decreases in phosphorylated PKA substrates compared with DMSO treated cells. Phosphorylated PKA substrates decreased by ∼51% (± 8%) in HeLa cells and by ∼44% (± 6%) in sheep PT cells (Fig. 3*A*). Similarly, to mock infected cells, BTV infected cells treated with the Dibutyryl-cAMP activator showed a significant increase in phosphorylated PKA substrates compared with DMSO-only treated cells, increasing by ∼26% (± 5%) in HeLa cells and by ∼33% (± 4%) in sheep PT cells (Fig. 3*A*).

In addition to these changes, we also observed changes in viral protein levels, including NS1 and NS2. When NS2 protein levels were normalized to host cell GAPDH levels via densitometry in replicate experiments, H89 treatment significantly decreased NS2 protein levels compared with the DMSO control (Fig. 3*A*). In HeLa cells, the viral NS2 protein levels decreased by ∼60% (± 11%) and by ∼64% (± 9%) in sheep PT cells following treatment of cells with the PKA inhibitor (Fig. 3*A*). Conversely, in cells treated with Dibutyryl-cAMP, the viral NS2 protein levels increased by ∼24% (± 12%) in HeLa cells and by ∼15% (± 6%) in sheep PT cells (Fig. 3*A*).

In parallel, to determine whether PKA inhibition also affected infectious virus production, virus titers of replicate lysates of infected cells were determined by plaque assays. At 12 h.p.i, the titer of the DMSO-only control was ∼3 × 10^4^ Pfu/ml, which decreased to ∼7.5 × 10^2^ Pfu/ml in the presence of 40 μm H89 and was∼2.7 × 10^4^ Pfu/ml in the presence of 1 mm Dibutyryl-cAMP (supplemental Fig. S10*A*).

At 18 h.p.i there is a significant increase of phosphorylated PKA substrates in infected HeLa and sheep PT cells when compared with mock infected cells (Fig. 3*B*), this observation agrees with the phosphoproteome data obtained (supplemental Table S3–S5). Like the data at 12 h.p.i., samples collected at 18 h.p.i (Fig. 3*B*) showed the effects of H89 and Dibutyryl-cAMP on the levels of phosphorylated PKA substrates normalized to host cell GAPDH levels in mock, or non-infected, HeLa or sheep PT cells. Mock infected cells treated with the H89 inhibitor showed a significant decrease in phosphorylated PKA substrates compared with DMSO-only treated cells, decreasing by ∼39% (± 12%) in HeLa cells and by ∼38% (± 17%) in sheep PT cells (Fig. 3*B*). Further, cells treated with the Dibutyryl-cAMP activator showed a significant increase in phosphorylated PKA substrates compared with DMSO-only treated cells, increasing by ∼26% (± 8%) in HeLa cells and by ∼24% (± 16%) in sheep PT cells (Fig. 3*B*).

BTV infection resulted in an increase of phosphorylated PKA substrates in HeLa cells by ∼18% (± 11%), and by ∼30% (± 15%) in sheep PT cells (Fig. 3*B*). Like the changes observed at 12 h.p.i, BTV infected cells treated with the H89 inhibitor showed significant decreases in phosphorylated PKA substrates compared with mock infected cells, decreasing by ∼36% (± 16%) in HeLa cells and by ∼51% (± 19%) in sheep PT cells (Fig. 3*B*). Like mock infected cells, BTV infected cells treated with the Dibutyryl-cAMP activator showed a significant increase in phosphorylated PKA substrates compared with DMSO-only treated cells, increasing by ∼45% (± 21%) in HeLa cells and by ∼37% (± 17%) in sheep PT cells (Fig. 3*B*). Following treatment with the PKA inhibitor, NS2 protein levels decreased significantly by ∼52% (± 14%) in HeLa cells and by ∼40% (± 17%) in sheep PT cells (Fig. 3*B*). In contrast, in cells treated with Dibutyryl-cAMP, the viral NS2 protein levels increased by ∼28% (± 15%) in HeLa cells and by ∼32% (± 15%) in sheep PT cells (Fig. 3*B*). Like 12 h.p.i, at 18 h.p.i, we also observed a decrease in virus titer when assaying replicate lysates via plaque assay. The titer of the DMSO-only control was ∼2 × 10^5^ Pfu/ml, decreasing to ∼2 × 10^3^ Pfu/ml at 40 μm H89 and was∼2.7 × 10^4^ Pfu/ml in the presence of 1 mm Dibutyryl-cAMP (supplemental Fig. S10*B*).

##### Decreases or Increases in PKA-dependent Phosphorylated Substrate Levels Prior To Infection Do Not Affect BTV Replication

**Fig. 4.**
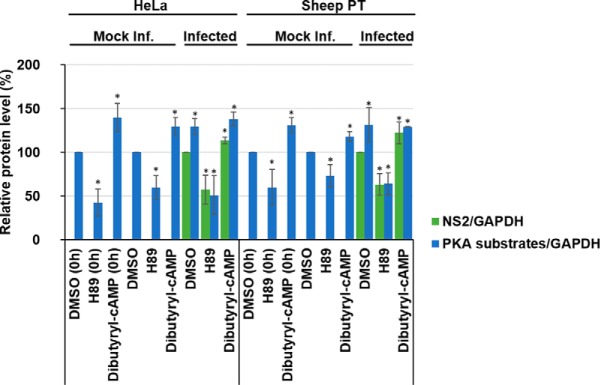
**Decreases or increases in PKA-dependent phosphorylated substrate levels prior to infection do not affect BTV replication.** HeLa and sheep PT cells were treated with 40 μm H89 or 1 mm Dibutyryl-cAMP for 1 h prior to infection with BTV1 (MOI = 5) and harvested 18 h.p.i. As controls, mock infected and DMSO treated cells were included. Samples were analyzed by Western blot densitometry analysis of phosphorylated-PKA substrates and NS2 are shown. Results are expressed as the percentage of protein levels as indicated. Error bars represent the S.D. values of stimulations from three independent experiments. A star (*) denotes a significant difference from control (*p* < 0.05).

Because our data showed that PKA inhibition decreases BTV replication and PKA activation increased BTV replication (Fig 3*A* and 3*B*) following infection, we wanted to examine whether depletion or enrichment of PKA phosphorylated substrates prior to virus entry could further decrease or increase virus replication by influencing virus entry (Fig. 4). An aspect that had previously been documented for HCV (17). Therefore, HeLa and sheep PT cells were pre-treated with DMSO, 40 μm H89 or 1 mm Dibutyryl-cAMP for 1 h prior to infection. Cell lysates were subsequently analyzed by Western blot analysis and representative Western blots are shown (supplemental Fig. S6).

Densitometry quantification of Western blots confirmed that at the time of infection (0 h), phosphorylated PKA substrate levels had significantly decreased following treatment with H89 by ∼58% (± 15%) in HeLa cells and by ∼40% (± 20%) in sheep PT cells. Cells treated with the Dibutyryl-cAMP activator showed a significant increase in phosphorylated PKA substrates compared with DMSO-only treated cells, increasing by ∼40% (± 16%) in HeLa cells and by ∼31% (± 9%) in sheep PT cells (Fig. 4). These data confirmed the respective depletion and enrichment of phosphorylated substrate prior to infection (Fig. 4).

At 18 h.p.i there is a significant increase of phosphorylated PKA substrates in infected HeLa and sheep PT cells when compared with mock infected cells (Fig. 4). Phosphorylated PKA substrates in infected HeLa cells increased by ∼30% (± 10%) and by ∼32% (± 19%) in sheep PT cells (Fig. 4). BTV infected cells treated with the H89 inhibitor showed significant decreases in phosphorylated PKA substrates, decreasing by ∼51% (± 22%) in HeLa cells, and by ∼36% (± 12%) in sheep PT cells (Fig. 4). Cells treated with the Dibutyryl-cAMP activator showed a significant increase in phosphorylated PKA substrates compared with DMSO-only treated cells, increasing by ∼38% (± 8%) in HeLa cells and by ∼29% (± 1%) in sheep PT cells (Fig. 4). Following treatment with the PKA inhibitor prior to infection, NS2 protein levels decreased significantly by ∼43% (± 17%) in HeLa cells and by ∼37% (± 12%) in sheep PT cells (Fig. 4). Conversely, in cells treated with Dibutyryl-cAMP, the viral NS2 protein levels increased by ∼14% (± 4%) in HeLa cells and by ∼23% (± 12%) in sheep PT cells (Fig. 4).

Overall, these data from post (Fig. 3) and pretreated (Fig. 4) samples suggest that depleting or enriching cells of phosphorylated PKA substrates prior to infection provides no effect on the inhibition or enhancement of viral replication. This may also suggest that these phosphorylated PKA substrate are not essential during viral cell entry.

##### Depletion of PKA Phosphorylated Substrates Following Infection with AHSV Reduces Viral Replication, Although Enrichment of PKA Phosphorylated Substrates Enhances Viral Replication

To investigate whether the activation of PKA is a common phenomenon among orbiviruses, we conducted similar studies using African horse sickness virus (AHSV). HeLa cells that support AHSV infection and natural host cells, E. Derm cells, an equid derived dermal cell line, were infected with AHSV in parallel. Cells were treated 1 h.p.i with DMSO, 40 μm H89 or 1 mm Dibutyryl-cAMP and harvested 18 h.p.i. Cell lysates were analyzed by Western blot (supplemental Fig. S7*A*).

**Fig. 5.**
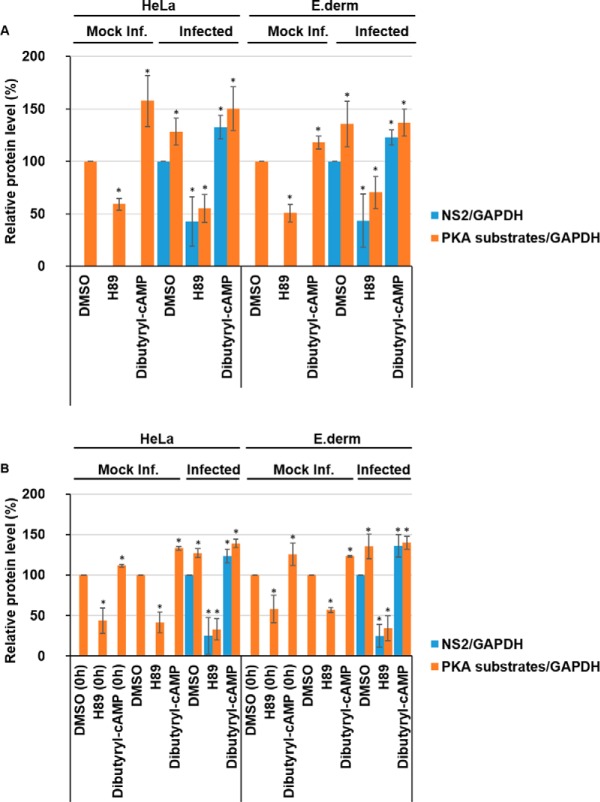
**Changes in the levels of PKA-dependent phosphorylated substrates post or prior to infection do not further affect AHSV replication.** HeLa and Equine dermal (E. Derm) cells were treated 1 h.p.i (*A*) or for 1 h prior (*B*) to infection with AHSV1 (MOI = 5) with 40 μm H89 or 1 mm Dibutyryl-cAMP and harvested 18 h.p.i. As controls, mock infected and DMSO treated cells were included. Densitometry analysis of phosphorylated-PKA substrates and NS2 are shown and the results presented as protein percentage, as indicated. Error bars represent the S.D. values of stimulations from three independent experiments. A star (*) denotes a significant difference from control (*p* < 0.05).

Quantification of Western blots by densitometry validated the effects of H89 and Dibutyryl-cAMP on the levels of phosphorylated PKA substrates normalized to host cell GAPDH levels in mock, or non-infected, HeLa or E. Derm cells (Fig 5*A*). Mock infected cells treated with the H89 inhibitor showed a significant decrease in phosphorylated PKA substrates compared with DMSO-only treated cells, decreasing by ∼41% (± 6%) in HeLa cells and by ∼49% (± 8%) in E. Derm cells. Cells treated with the Dibutyryl-cAMP activator showed a significant increase in phosphorylated PKA substrates compared with DMSO-only treated cells, increasing by ∼57% (± 24%) in HeLa cells and by ∼36% (± 13%) in E. Derm cells.

AHSV infection resulted in an increase of phosphorylated PKA substrates in HeLa cells by ∼29% (± 12%), and by ∼36% (± 21%) in E. Derm cells (Fig. 5*A*). Infected cells treated with the H89 inhibitor showed significant decreases in phosphorylated PKA substrates compared with mock infected cells, decreasing by ∼45% (± 13%) in HeLa cells, and by ∼30% (± 15%) in E. Derm cells. Infected cells treated with the Dibutyryl-cAMP activator showed a significant increase in phosphorylated PKA substrates compared with DMSO-only treated cells. Phosphorylated PKA substrates increased by ∼50% (± 20%) in HeLa cells and by ∼37% (± 13%) in E. Derm cells. Following treatment with the PKA inhibitor, NS2 protein levels decreased significantly by ∼58% (± 23%) in HeLa cells and by ∼57% (± 25%) in E. Derm cells. Whereas in cells treated with Dibutyryl-cAMP, the viral NS2 protein levels increased by ∼33% (± 11%) in HeLa cells and by ∼23% (± 7%) in E. Derm cells. These effects on viral replication were further validated by determining virus titer following treatment with H89 and Dibutyryl-cAMP. 18 h.p.i, the titer of the DMSO-only control was ∼5.5 × 10^5^ Pfu/ml, decreasing to ∼2.1 × 10^3^ Pfu/ml at 40 μm H89 and was ∼2.5 × 10^5^ Pfu/ml in the presence of 1 mm Dibutyryl-cAMP (supplemental Fig. S10*C*)

##### Changes in PKA-dependent phosphorylated substrate levels prior to infection do not further impair or enhance AHSV replication

To test whether our observations using BTV were further comparable to AHSV, we examined whether depletion or enrichment of PKA phosphorylated substrates prior to virus entry could further decrease or increase virus replication (Fig. 5*B*). Therefore, HeLa and E. Derm cells were pretreated with DMSO, 40 μm H89 or 1 mm Dibutyryl-cAMP for 1 h prior to infection and cell lysates were subsequently analyzed by Western blot (supplemental Fig. S7*B*).

As expected at the time of infection (0 h), phosphorylated PKA substrate levels had significantly decreased following a 1 h treatment with H89 by ∼56% (± 15%) in HeLa cells and by ∼42% (± 17%) in E. Derm cells (Fig. 5*B*). Cells treated with the Dibutyryl-cAMP activator showed a significant increase in phosphorylated PKA substrates compared with DMSO-only treated cells, increasing by ∼12% (± 2%) in HeLa cells and by ∼26% (± 14%) in E. Derm cells. This confirmed the respective depletion and enrichment of phosphorylated substrate prior to infection.

At 18 h.p.i there is a significant increase of phosphorylated PKA substrates in infected HeLa and E. Derm cells when compared with mock infected cells (Fig. 5*B*). Phosphorylated PKA substrates in infected HeLa cells significantly increased by ∼27% (± 6%) and by ∼36% (± 15%) in E. Derm cells. AHSV infected cells treated with the H89 inhibitor showed significant decreases in phosphorylated PKA substrates, decreasing by ∼67% (± 13%) in HeLa cells, and by ∼66% (± 15%) in E. Derm cells (Fig. 5*B*). Concurrently, infected cells treated with the Dibutyryl-cAMP activator showed a significant increase in phosphorylated PKA substrates compared with DMSO-only treated cells, increasing by ∼39% (± 5%) in HeLa cells and by ∼40% (±8%) in E. Derm cells. Following treatment with the PKA inhibitor prior to infection, NS2 protein levels decreased significantly by ∼75% (± 22%) in HeLa cells and by ∼75% (± 14%) in E. Derm cells. Cells treated with Dibutyryl-cAMP, the viral NS2 protein levels increased by ∼24% (± 8%) in HeLa cells and by ∼36% (± 14%) in E. Derm cells (Fig. 5*B*). These data show that BTV and AHSV share a similar response profile, this may suggest that these phosphorylated PKA substrate are not essential during viral cell entry or early stages of virus replication.

##### Both BTV and AHSV Infection Increase Phosphorylation of AKT Substrates

AKT pathway activity featured in our phosphoproteomics analysis, specifically via the Motif-X analysis (supplemental Table S6), a pathway that was previous reported to be involved in the BTV life-cycle (20), we thus proceeded to use this for further validation of our phosphoproteomics analysis. HeLa and sheep PT cells were infected with BTV whereas HeLa and E. Derm cells were infected with AHSV. Cell lysates were subsequently analyzed by Western blot analysis and representative Western blots are shown for BTV and AHSV. To determine the effects of inhibition of AKT activity and to validate down-regulation of the AKT pathway, cells were treated 1 h.p.i with 4 μm Akt Inhibitor VIII (AKT VIII) and harvested 18 h.p.i. (supplemental Fig. S8*A* and S8*B*).

**Fig. 6.**
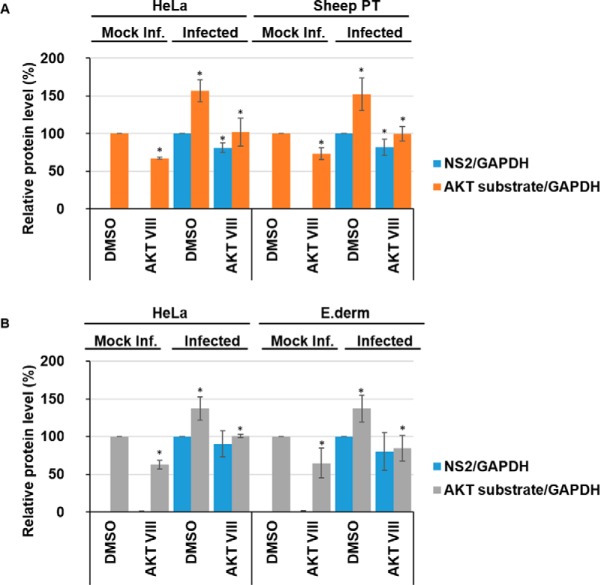
**BTV1 and AHSV1 increase AKT-dependent phosphorylated substrates.** HeLa and sheep PT cells infected with BTV1 (MOI = 5) (*A*) or HeLa and Equine dermal (E. Derm) cells infected with AHSV1 (MOI = 5) (*B*) were treated 1 h.p.i with 4 μm Akt Inhibitor VIII (AKT VIII) and harvested 18 h.p.i. Densitometry analysis of phosphorylated-AKT substrates and NS2 are presented as relative protein level percentage. Error bars represent the S.D. values of stimulations from three independent experiments. A star (*) denotes a significant difference from control (*p* < 0.05).

Mock infected cells treated with the Akt Inhibitor VIII exhibited a significant decrease in phosphorylated AKT substrates compared with DMSO-only treated cells, decreasing by ∼33% (± 2%) in HeLa cells and by ∼27% (± 8%) in sheep PT cells (Fig. 6*A*). BTV infection resulted in an increase of phosphorylated AKT substrates in HeLa cells by ∼57% (± 15%), and by ∼52% (± 21%) in sheep PT cells (Fig. 6*A*). Akt Inhibitor VIII treatment could significantly decrease the levels of phosphorylated AKT substrate in DMSO treated infected cells, decreasing by ∼55% (± 19%) in HeLa cells and by ∼52% (± 10%) in sheep PT cells. Further, NS2 protein levels decreased by ∼19% (± 6%) in HeLa cells and by ∼18% (± 11%) in sheep PT cells (Fig. 6*A*). However, we did not observe any concomitant decrease in virus titer in the presence of the AKT inhibitor (supplemental Fig. S10*D*).

Similarly, we then proceeded to determine whether AHSV infections would elicit a similar response. As we had done for BTV, we validated the effects of Akt Inhibitor VIII on the levels of phosphorylated AKT substrates normalized to host cell GAPDH levels in mock, or non-infected, HeLa and E. Derm cells. Mock infected cells treated with the Akt Inhibitor VIII showed a significant decrease in phosphorylated AKT substrates compared with DMSO-only treated cells, decreasing by ∼37% (± 6%) in HeLa cells and by ∼35% (± 19%) in E. Derm cells (Fig. 6*B*).

Similarly, AHSV infection resulted in an increase of phosphorylated AKT substrates in HeLa cells by ∼38% (± 16%), and by ∼37% (± 18%) in E. Derm cells (Fig. 6*B*). Akt Inhibitor VIII treatment could significantly decrease the levels of phosphorylated AKT substrate in DMSO treated infected cells, decreasing by ∼37% (± 2%) in HeLa cells and by ∼53% (± 17%) in E. Derm cells. Following treatment with Akt Inhibitor VIII, NS2 protein levels did not significantly decrease in either HeLa cells nor in E. Derm cells and no observable decrease in virus titer in the presence of the AKT inhibitor was detected (supplemental Fig. S10*E*).

**Fig. 7.**
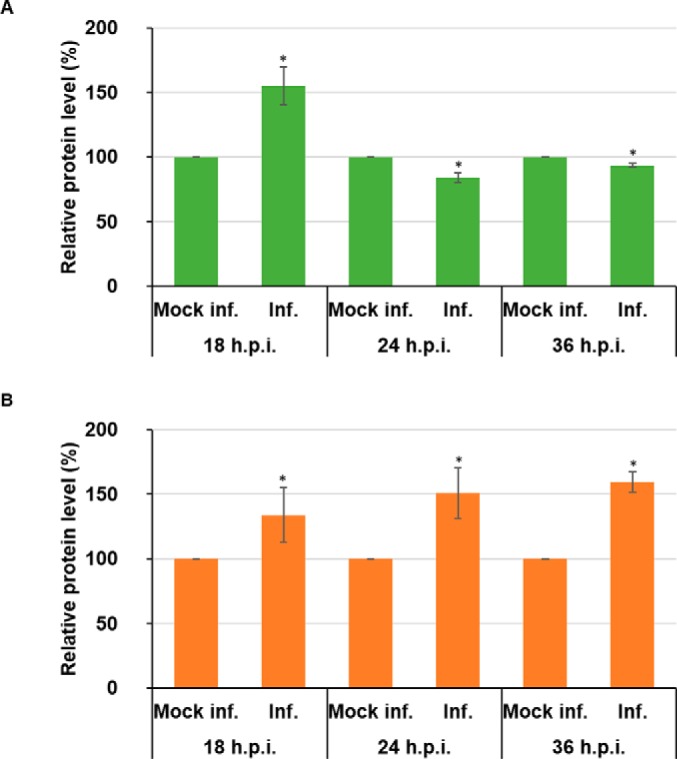
**AKT-dependent phosphorylated substrates decrease in BTV1 infected cells but remain elevated in AHSV1 infected cells between 18 and 36 h.p.i. HeLa cells infected with BTV1 (MOI = 5) (*A*) or HeLa cells infected with AHSV1 (MOI = 5) (*B*) were harvested 18, 24, and 36 h.p.i.** Densitometry analysis of phosphorylated-AKT substrates are expressed as relative protein level as indicated. Error bars represent the S.D. values of stimulations from three independent experiments. A star (*) denotes a significant difference from control (*p* < 0.05).

However, a previous body of work had reported that AKT pathway activity decreased at 36 h.p.i., we set out to investigate this potential incongruity. We carried out a time-course experiment, harvesting BTV and AHSV infected HeLa cells at 18 h.p.i, 24 h.p.i and 36 h.p.i. Cell lysates were analyzed by Western blot (supplemental Fig. S9). At 18 h.p.i there is a significant increase of phosphorylated AKT substrates in infected HeLa cells when compared with mock infected cells (∼55% (± 15%)). However, the levels of phosphorylated AKT substrates significantly decreased at 24 h.p.i and 36 h.p.i by ∼71% (± 4%) and by ∼62% (± 2%), respectively, compared with infected cells analyses at 18 h.p.i. (Fig. 7*A*).

AHSV showed a different pattern in its profile of phosphorylated AKT substrates. At 18 h.p.i there is a significant increase of phosphorylated AKT substrates in infected HeLa cells (∼34% (± 21%)). However, unlike BTV, we did not observe a similar decrease, at 24 h.p.i and 36 h.p.i phosphorylated substrate levels remained ∼51% (± 20%) and ∼59% (± 8%), respectively, above the levels of mock infected cells (Fig. 7*B*).

Together, these data suggest that the activation of the AKT pathway occurs during infection by both orbiviruses. However, although AKT pathway activity significantly decreases between 18 h.p.i and 36 h.p.i. for BTV, pathway activity remains elevated for AHSV throughout the time points tested.

## DISCUSSION

Reversible protein phosphorylation is the most pervasive control and regulatory mechanism within cells to facilitate the continual adjustment of catabolic, anabolic and signal transduction events to maintain cellular equilibrium (36). The number of potential candidates is significant, given that it has been estimated that up to 30% of expressed proteins contain at least one covalently bound phosphate (37). This dynamic and interconnected web of phosphorylated and dephosphorylated proteins is encapsulated within a cells phosphoproteome and the examination for exploitation by viruses highly pertinent. Changes in the phosphoproteome of host cells during infection provides an insight into the pathways exploited or suppressed by a virus to facilitate successful propagation, and its analysis may yield hitherto unknown or exploited opportunities for therapeutic interventions. Previously, several other viruses have been studied in this way, including influenza A virus (38), influenza B (39) and West Nile virus (40). However, although proteomic studies have been carried out for the family *Reoviridae*, using mammalian reoviruses (MRV) (41), to date no global phosphoproteomic studies have interrogated BTV infected cells prior to this study.

Given that BTV is the prototype member of the *Orbivirus* genus in the *Reoviridae* family, we set out to characterize the phosphoproteome of HeLa cells infected with BTV at both 12 h.p.i and 18 h.p.i. During the subsequent analysis, we confirmed that casein kinase 2 was active, this finding supports previous observations that delineated the significance of this kinase and its activity for the phosphorylation of BTV NS2 (13, 14). Furthermore, we could validate PKA as a novel host cell factor utilized by both BTV and AHSV, whose target substrates showed increased phosphorylation during infection at 18 h.p.i. Functional studies using a PKA inhibitor or a PKA activator showed impaired or enhanced viral replication, respectively, in both HeLa and sheep PT cells, a natural host derived cell line. Concurrently, these results could be replicated using AHSV, suggesting a broader role for PKA within the *Orbivirus* genus. Inhibition of PKA prior to infection did not result in a further reduction of viral replication, this contrasts with observations made during studies of HCV, where inhibition of PKA led to a reduction in viral entry (17).

The modulation of the PKA pathway by BTV may correlate to the role the PKA pathway plays in cell proliferation (42, 43) by cell cycle arrest via p38 MAPK (44), impeding this process for the reallocation of cellular resources into virus production. In a recent study, the autophagy signaling network initiated by BTV infection was examined at 24 h.p.i and 36 h.p.i (20). The phosphorylation candidates that were examined, included extra-cellular signal-regulated kinase 1/2 (ERK1/2), p70S6K, mammalian target of rapamycin (mTOR) and AKT. Although our study examines the phosphoproteome prior to these time points, to avoid early CPE or cell death, it has allowed us to compare our observations with these later time points.

In this context, BTV mediated PKA activity could result in the inhibition of extracellular signal-regulated kinase 1/2 (ERK1/2) activation (45, 46) which under certain circumstances has anti-apoptotic effects (47). Our phosphoproteomic analysis found that ERK1/2 activity was decreased at 18 h.p.i. ERK1/2 signaling pathway can promote cell survival by mediating the phosphorylation of Bad and/or Bim through multiple mechanisms, promoting cell proliferation and reducing the sensitivity of cells to apoptosis (48). The decreased activity of ERK1/2 suggests an increased sensitivity of the infected cells to apoptosis, which has previously been shown to occur in BTV infected mammalian cells (49). However, with regards to ERK1/2, Lv *et al.* found that there was no significant difference between mock and BTV infected cells 36 h.p.i (20).

Our phosphoproteomic analysis also found that p70S6K was active at both 12 h.p.i and 18 h.p.i. As p70S6K functions to regulate protein synthesis (50, 51), this suggests that translation and protein synthesis could be enhanced within the infected cells at these times. Furthermore, it may contribute to the inhibition of apoptosis by the phosphorylation of the BCL2 associated agonist of cell death (Bad) protein (52). Interestingly, p70S6K phosphorylation was found to be diminished with increasing infection time, being 4.5 times lower at 36 h.p.i (20), This, however, could be related to a host cell translational shut-down at such a late time point (11).

In trying to dissect the autophagy induction network utilized by BTV, it has been found that at 36 h.p.i, AKT diminished (20). Our Motif-X analysis led us to examine changes in AKT substrate phosphorylation. Our analysis, using Western blots, showed significant increases in phosphorylate substrates at 18 h.p.i. However, a time-course experiment encompassing 18 h.p.i., 24 h.p.i., and 36 h.p.i revealed the subsequent decrease in phosphorylated AKT substrates during BTV infection. These changes highlight the temporal and dynamic nature of phosphoproteome during BTV infection, with AKT being active during the early phase of infection before diminishing in the later stages. Strikingly, AHSV did not portray a similarly dynamic nature.

**Fig. 8.**
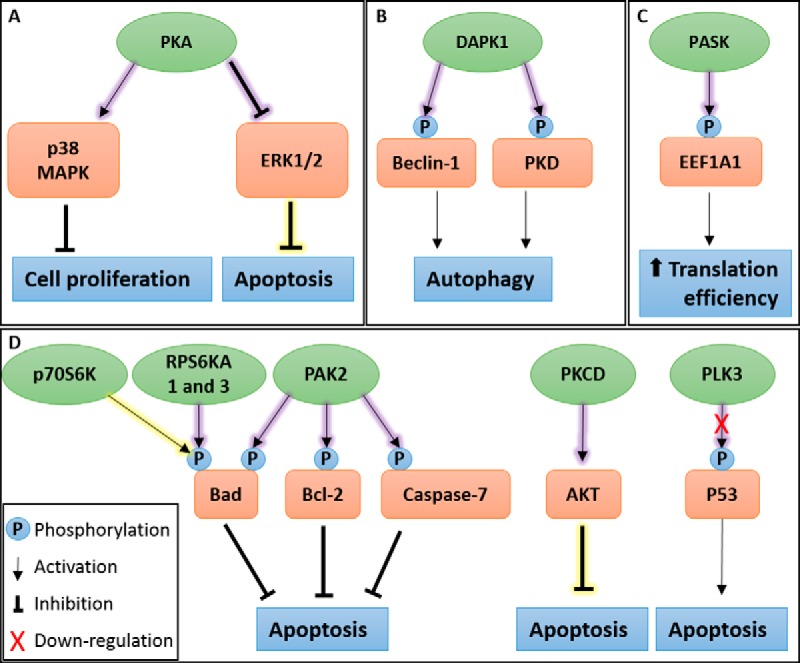
**A schematic representation of pathways summary showing identified kinases in BTV infected cells.** *A*, Protein kinase A (PKA) activity may contribute to apoptosis inhibition and arrest of cell proliferation, extending the duration of viral replication. Validation experiments confirmed the importance of PKA activity for BTV replication. *B*, BTV infection induces autophagy. Death-associated protein kinase 1 (DAPK1) activity was identified and may contribute to autophagy induction during BTV infection. *C*, PAS Domain Containing Serine/Threonine Kinase (PASK) activity increased during BTV infection, possibly increasing translation efficiency during viral protein synthesis. *D*, Apoptosis is known to be controlled during BTV infection. Several kinases were identified as regulated by BTV infection which may contribute to the inhibition of apoptosis, these include ribosomal protein S6 kinase beta-1 (p70S6K), ribosomal protein S6 kinase A1 and 3 (RPS6KA1 and 3), p21-activated protein kinase 2 (PAK2), protein kinase C delta type (PKCD) activation and polo-like kinase 3 (PLK3). Inset shows various symbols: phosphorylation events, pathway activation, inhibition and down-regulation. A yellow shadow shows known kinase activity and a purple shadow depicts novel kinase activity.

Furthermore, the prospective novel kinases that this study identified, including PAK2, PKCD, PASK, DAPK1, RPS6KA1 and 3, and PLK3, could also contribute to virus propagation (Fig. 8). By activating PAK2, we hypothesis that BTV may promote cell survival via the phosphorylation of caspase-7 (53), Bad and B-Cell CLL/Lymphoma 2 (Bcl-2) thereby blocking apoptosis (54), which could allow for the extending of the duration of virus replication within a cell. Similarly, the upregulation of PKCD may also contribute to cell survival via the AKT pathway (55). Furthermore, we speculate that activation of PASK may enhance viral protein translation by increasing translation efficiency through the phosphorylation of eukaryotic translation elongation factor 1 alpha 1 (EEF1A1) (56). Upregulation of DAPK1, although implicated in pathways leading to apoptosis (57), has also been shown to induce and regulate autophagy (58). Autophagy has previously been described in a proviral context with regards to BTV replication (59). It could be hypothesized that DAPK1 activity may contribute to the induction of autophagy by phosphorylation of protein kinase D (PKD) (60) and the direct phosphorylation of Beclin1 (61), both of which act at the induction stage of autophagosome nucleation via modulating the Vps34 class III phosphatidyl inositol 3-kinase complex (58). Like PAK2 and PKCD, activation of RPS6KA1 and 3 could also contribute to cell survival and extending the duration that BTV can replicate within a cell. Specifically, through the phosphorylation of BAD, which suppresses its pro-apoptotic function (62, 63). It can also be hypothesized that the down-regulation of PLK3 may further contribute to interfering with the induction of apoptosis via a decreased ability to mediate P53 phosphorylation (64).

Interestingly, several of the novel kinases we identified, and the pathways they regulate, appear to suppress apoptosis at the times this study was carried out (12 h.p.i and 18 h.p.i). These observations agree with a previous study, showing that the later stages of apoptosis, denoted by the cleavage and inactivation of poly (ADP-ribose) polymerase (PARP), only become detectable 24 h.p.i and onwards (65).

In conclusion, it is noteworthy that both BTV and AHSV, with their distinct host range, require certain common host factors (PKA), suggesting Orbiviruses may share some key host factors and pathways during their infection. However, these studies also showed their distinctiveness with regards to their respective temporal phosphorylated AKT substrate profiles. Interestingly, our phosphoproteomic analysis also highlighted differences that might be relevant at later stages in virus replication and pathogenesis.

## DATA AVAILABILITY

The mass spectrometry proteomics data have been deposited to the ProteomeXchange Consortium via the PRIDE partner repository with the dataset identifier PXD005550.

## Supplementary Material

Supplemental Data

## Footnotes

^1^ The abbreviations used are:

BTV: Bluetongue virus
AHSV: African horse sickness virus
PKA: protein kinase A
h.p.i.: hours post infection.
