# Supplementary material for: Phosphoproteomic Analysis Reveals the Importance of Kinase Regulation During Orbivirus Infection
Source: Mol Cell Proteomics. 2017 Aug 29;16(11):1990–2005. doi: 10.1074/mcp.M117.067355 (PMC5672004; doi:10.1074/mcp.M117.067355)
Supplement: Supplemental Data [file supp_16_11_1990__index.html]

Phosphoproteomic analysis reveals the importance of kinase regulation during orbivirus infection — Phosphoproteomic Analysis Reveals the Importance of Kinase Regulation During Orbivirus Infection — Phosphoproteomic Analysis of BTV Infected Cells — Supplemental Data 

# Phosphoproteomic Analysis Reveals the Importance of Kinase Regulation During Orbivirus Infection

## Supplemental Data

- Supplementary Figures (.pdf, 932 KB) - Supplementary Figures
- Supplementary Figure legends (.pdf, 237 KB) - Supplementary Figure legends
- Table S1 phosphoproteins (.xlsx, 428 KB) - Table S1 phosphoproteins
- Table S2 Phosphopeptides (.xlsx, 992 KB) - Table S2 Phosphopeptides
- Table S3 phosphosites (.xlsx, 429 KB) - Table S3 phosphosites
- Table S4 Regulated phosphoprotein pep and sites only (.xlsx, 778 KB) - Table S4 Regulated phosphoprotein pep and sites only
- Table S5 GO analysis of regulated phosphoproteins (.xlsx, 108 KB) - Table S5 GO analysis of regulated phosphoproteins
- Table S6 Motif-X analysis (.xlsx, 52 KB) - Table S6 Motif-X analysis
- Table S7 Phoxtrack analysis (.xlsx, 119 KB) - Table S7 Phoxtrack analysis
