# Supplementary material for: Phosphoproteomic Analysis Reveals the Importance of Kinase Regulation During Orbivirus Infection
Source: Mol Cell Proteomics. 2017 Aug 29;16(11):1990–2005. doi: 10.1074/mcp.M117.067355 (PMC5672004; doi:10.1074/mcp.M117.067355)
Supplement: Supplemental Data [file 10.1074_M117.067355_mcp.M117.067355-1.pdf]

Figure S1

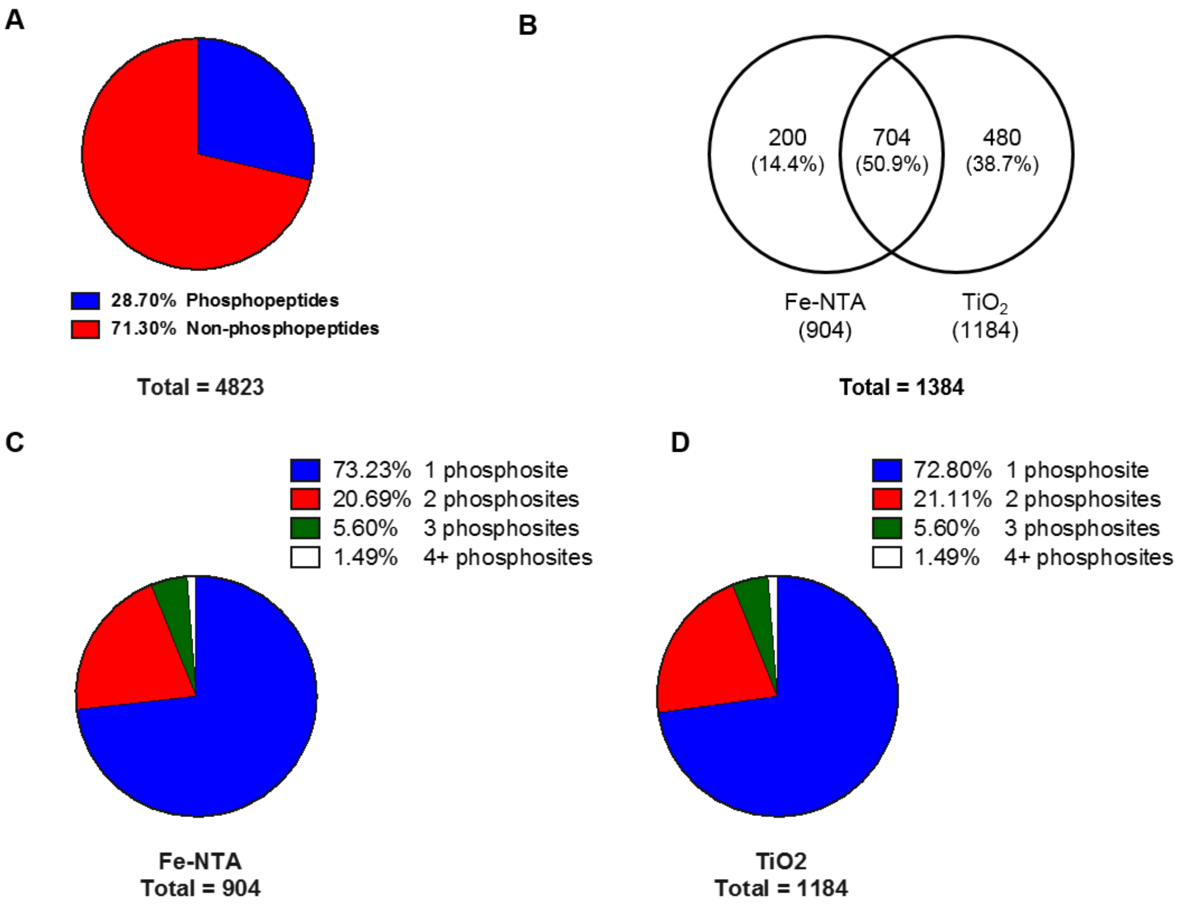
A
28.70% Phosphopeptides
71.30% Non-phosphopeptides
Total = 4823
B
200
(14.4%)
704
(50.9%)
480
(38.7%)
Fe-NTA
(904)
TiO_2
(1184)
Total = 1384
C
73.23% 1 phosphosite
20.69% 2 phosphosites
5.60% 3 phosphosites
1.49% 4+ phosphosites
Fe-NTA
Total = 904
D
72.80% 1 phosphosite
21.11% 2 phosphosites
5.60% 3 phosphosites
1.49% 4+ phosphosites
TiO2
Total = 1184

Figure S2

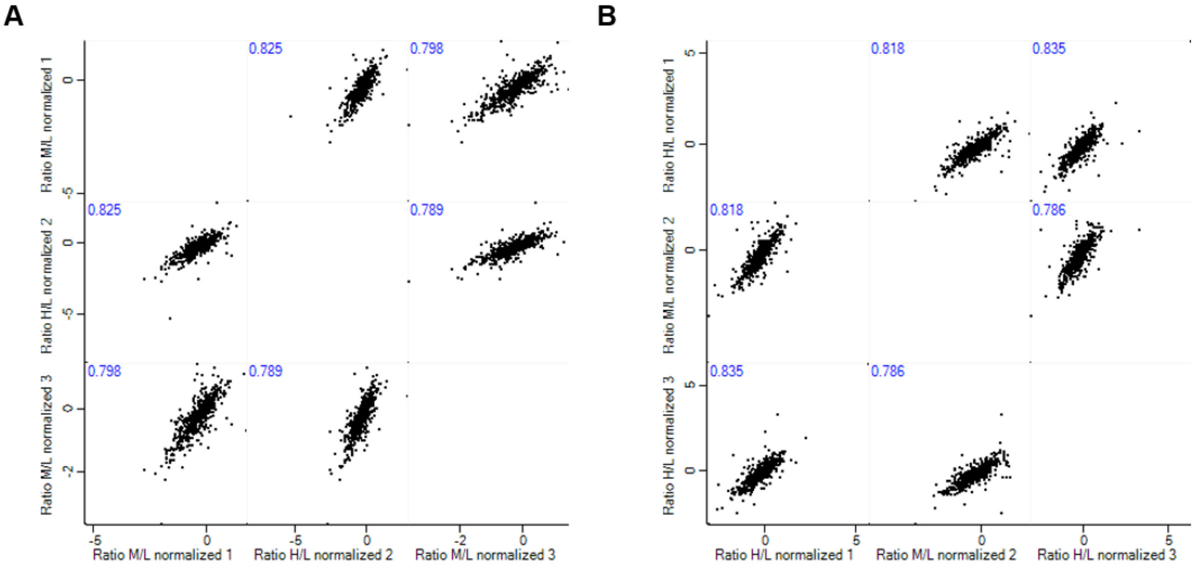

A
B
Ratio M/L normalized 1
Ratio H/L normalized 2
Ratio M/L normalized 3
0.825
0.798
0.789
Ratio H/L normalized 1
Ratio M/L normalized 2
Ratio H/L normalized 3
0.818
0.835
0.786

Figure S3

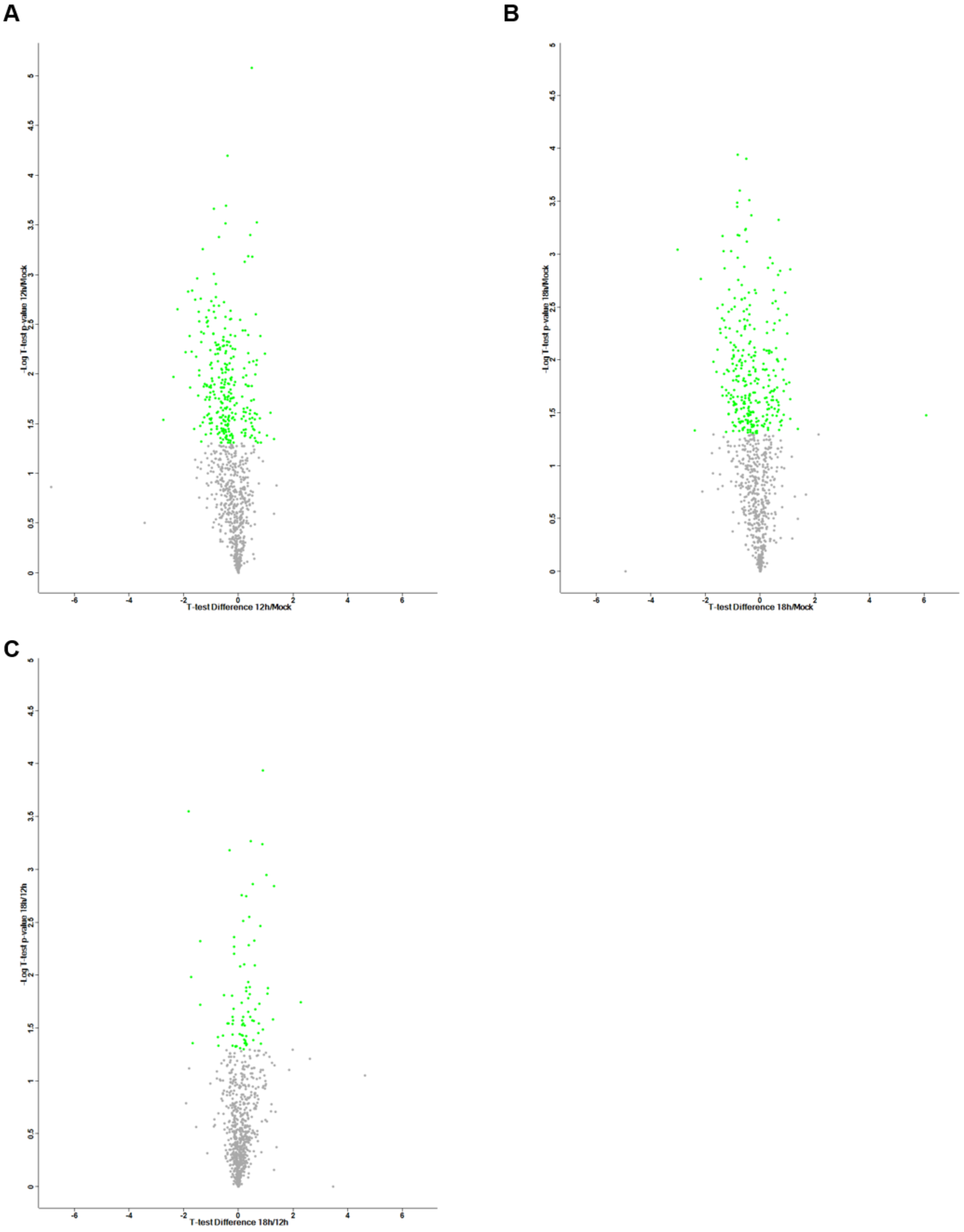
A
B
C
-Log T-test p-value 12h/Mock
T-test Difference 12h/Mock
-Log T-test p-value 18h/Mock
T-test Difference 18h/Mock
-Log T-test p-value 18h/12h
T-test Difference 18h/12h

Figure S4

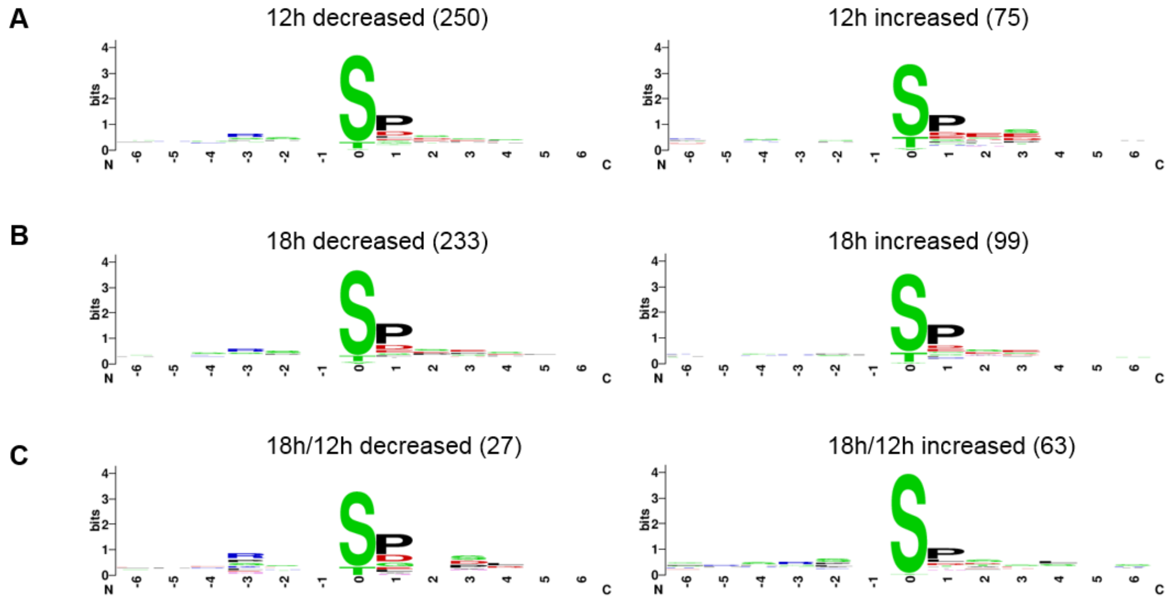
A
12h decreased (250)
12h increased (75)
B
18h decreased (233)
18h increased (99)
C
18h/12h decreased (27)
18h/12h increased (63)
bits
N
C

Figure S5

A

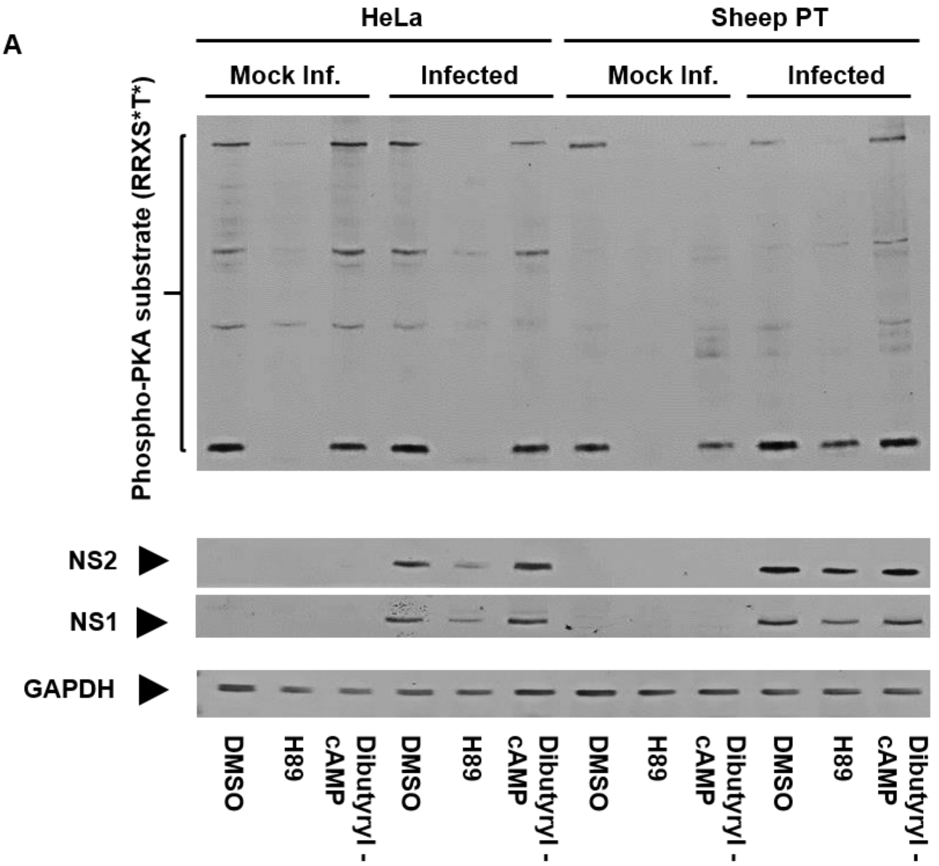
HeLa
Sheep PT
Mock Inf.
Infected
Mock Inf.
Infected
Phospho-PKA substrate (RRXS*T*)
NS2
NS1
GAPDH
DMSO
H89
Dibutyryl - cAMP
DMSO
H89
Dibutyryl - cAMP
DMSO
H89
Dibutyryl - cAMP
DMSO
H89
Dibutyryl - cAMP

B

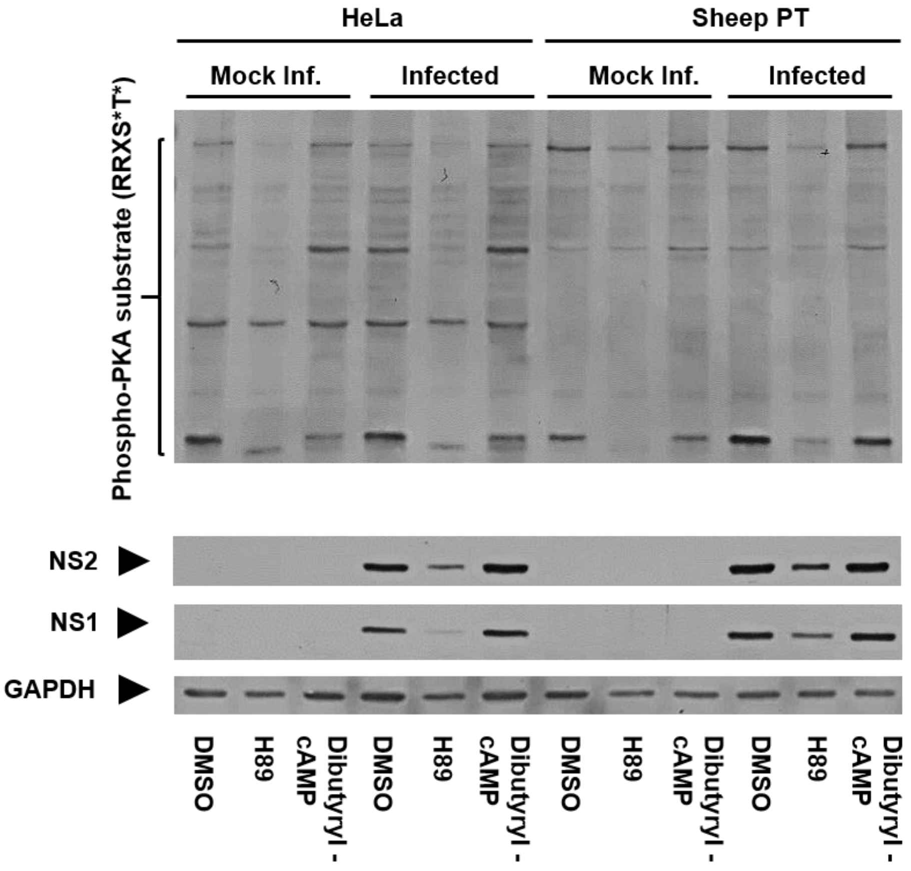
HeLa
Sheep PT
Mock Inf.
Infected
Mock Inf.
Infected
Phospho-PKA substrate (RRXS*T*)
NS2
NS1
GAPDH
DMSO
H89
Dibutyryl - cAMP
DMSO
H89
Dibutyryl - cAMP
DMSO
H89
Dibutyryl - cAMP
DMSO
H89
Dibutyryl - cAMP

Figure S6

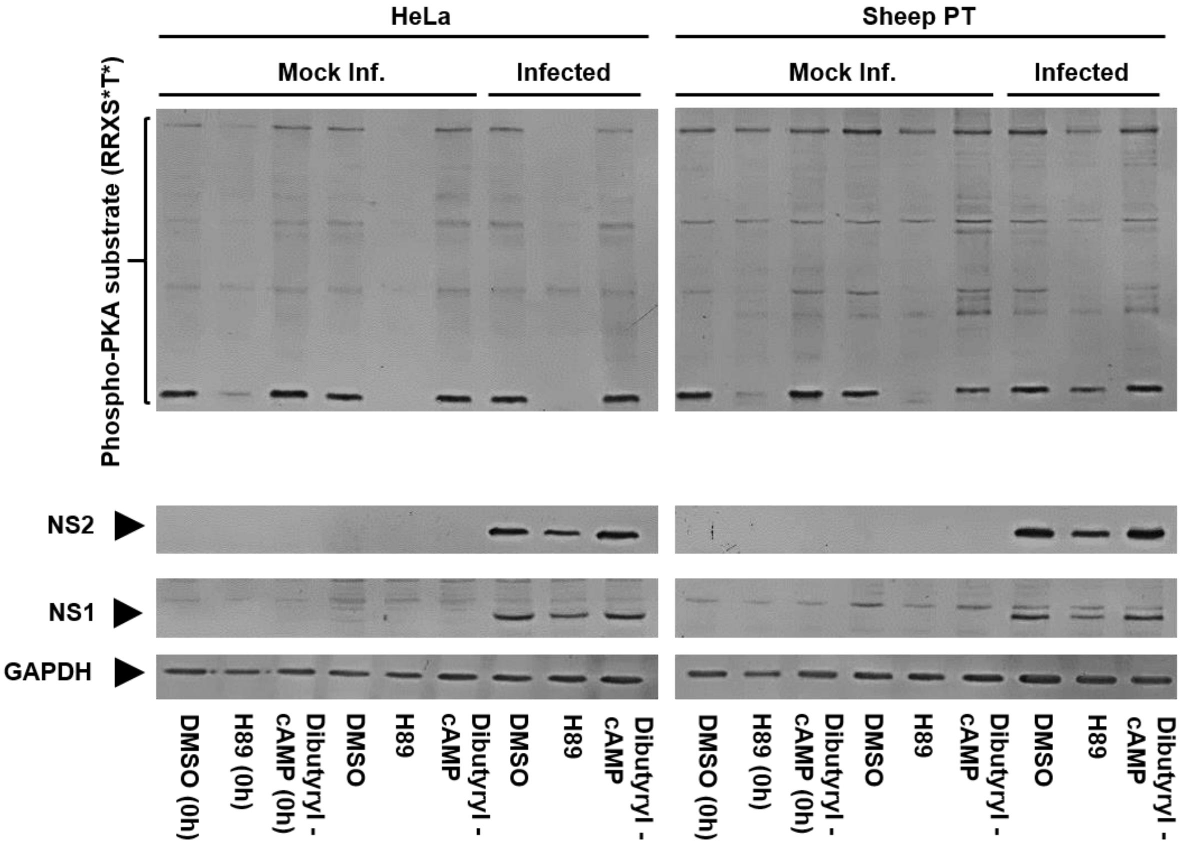

HeLa
Sheep PT
Mock Inf.
Infected
Mock Inf.
Infected
Phospho-PKA substrate (RRXS*T*)
NS2
NS1
GAPDH
DMSO (0h)
H89 (0h)
Dibutyryl - cAMP (0h)
DMSO
H89
Dibutyryl - cAMP
DMSO
H89
Dibutyryl - cAMP
DMSO (0h)
H89 (0h)
Dibutyryl - cAMP (0h)
DMSO
H89
Dibutyryl - cAMP
DMSO
H89
Dibutyryl - cAMP

Figure S7

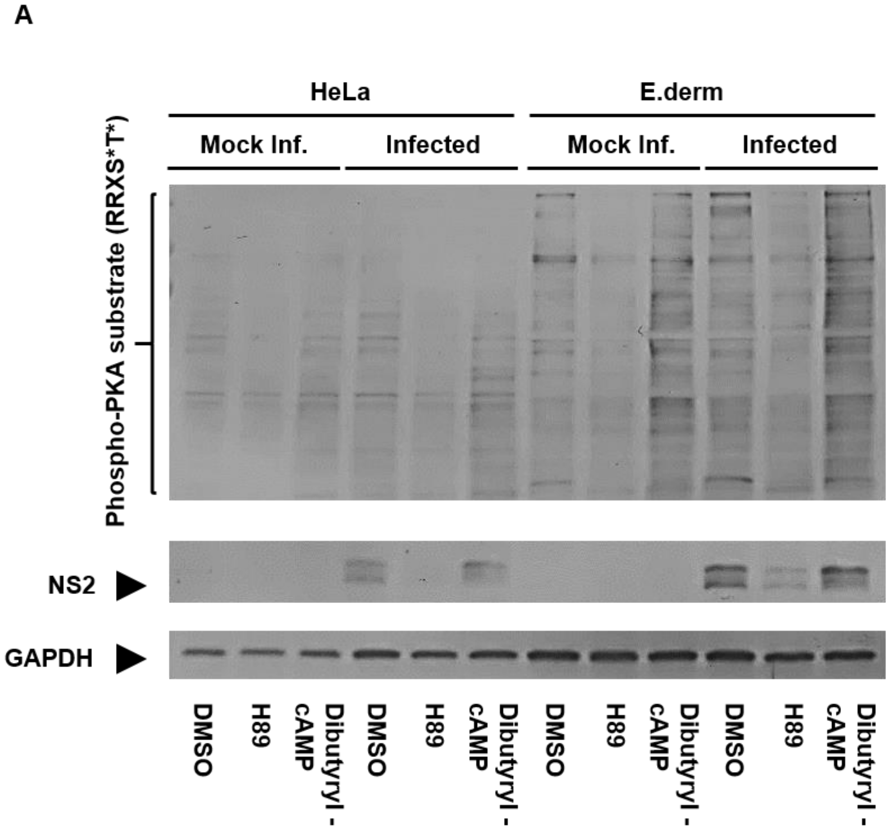
A
HeLa
E.derm
Mock Inf.
Infected
Mock Inf.
Infected
Phospho-PKA substrate (RRXS*T*)
NS2
GAPDH
DMSO
H89
Dibutyryl - cAMP
DMSO
H89
Dibutyryl - cAMP
DMSO
H89
Dibutyryl - cAMP
DMSO
H89
Dibutyryl - cAMP

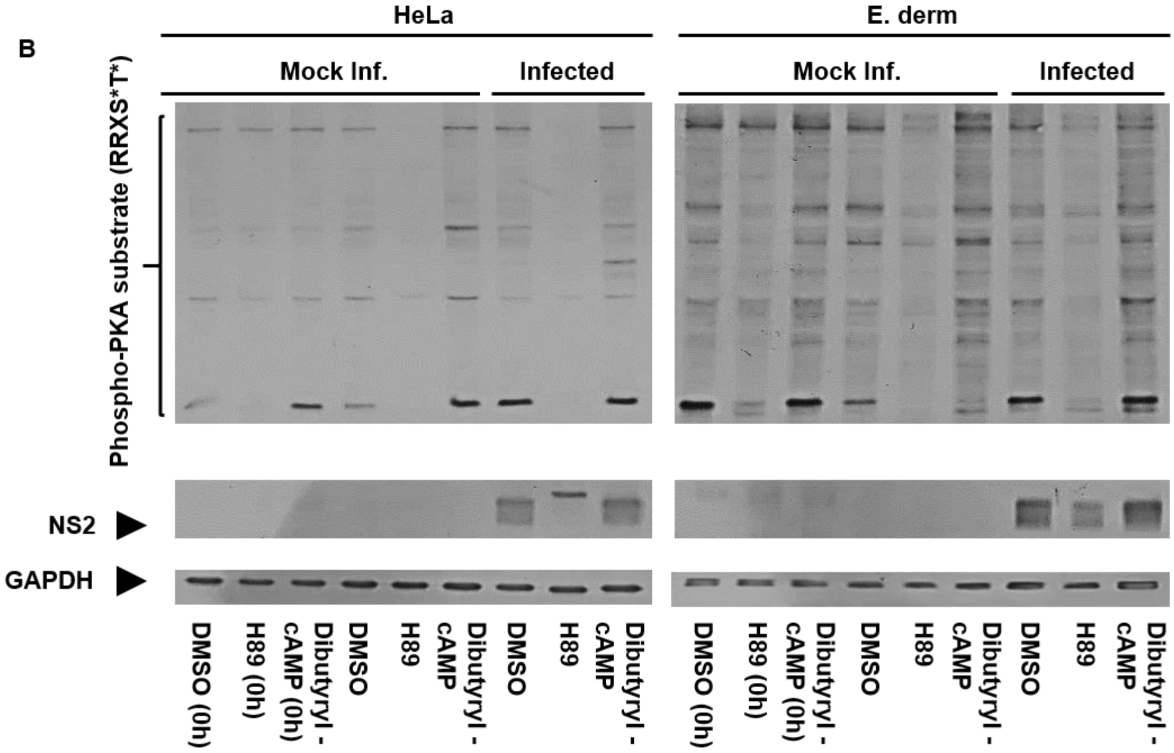
B
HeLa
E. derm
Mock Inf.
Infected
Mock Inf.
Infected
Phospho-PKA substrate (RRXS*T*)
NS2
GAPDH
DMSO (0h)
H89 (0h)
Dibutyryl - cAMP (0h)
DMSO
H89
Dibutyryl - cAMP
DMSO
H89
Dibutyryl - cAMP
DMSO (0h)
H89 (0h)
Dibutyryl - cAMP (0h)
DMSO
H89
Dibutyryl - cAMP
DMSO
H89
Dibutyryl - cAMP

Figure S8

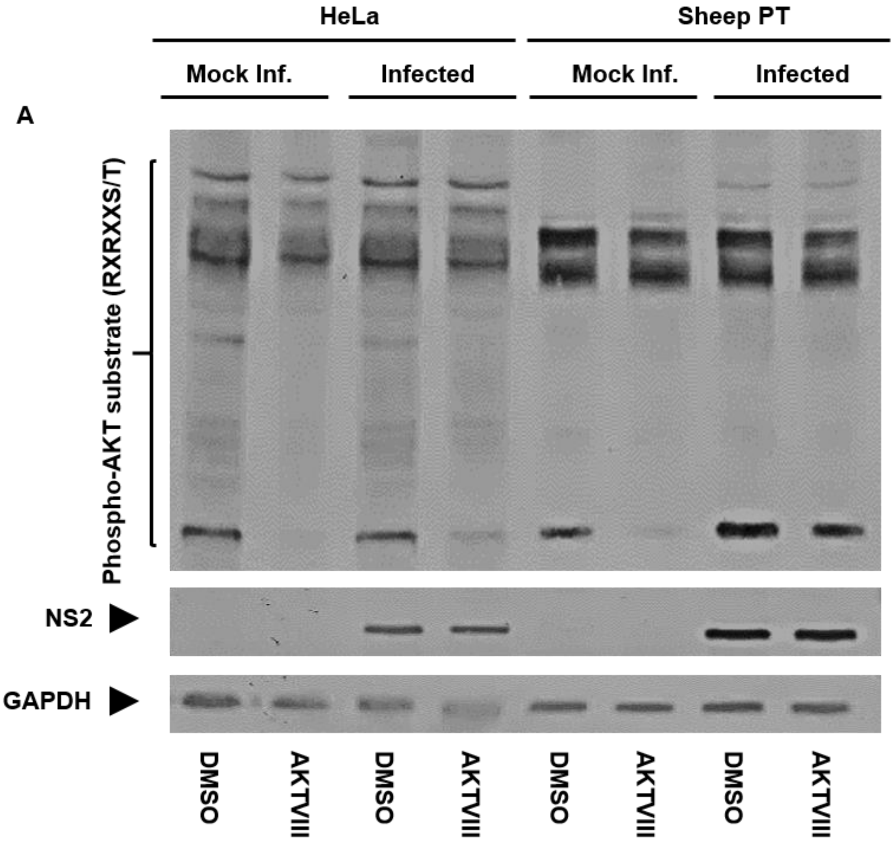
A
HeLa
Sheep PT
Mock Inf.
Infected
Mock Inf.
Infected
Phospho-AKT substrate (RXRXXS/T)
NS2
GAPDH
DMSO
AKTVIII
DMSO
AKTVIII
DMSO
AKTVIII
DMSO
AKTVIII

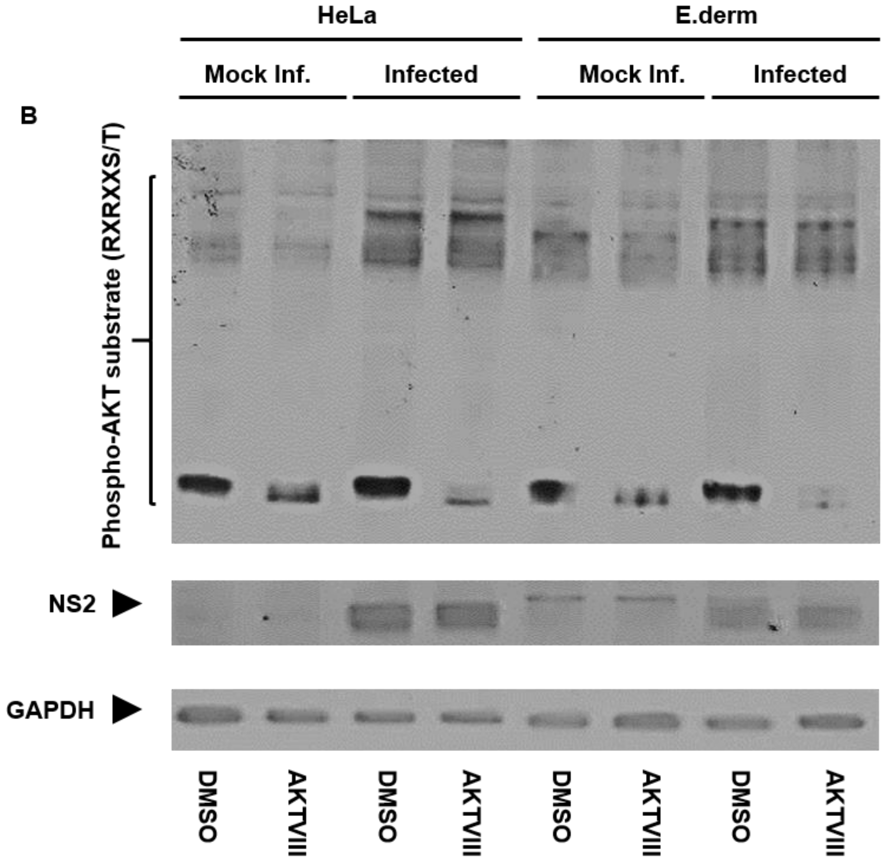
B
HeLa
E.derm
Mock Inf.
Infected
Mock Inf.
Infected
Phospho-AKT substrate (RXRXXS/T)
NS2
GAPDH
DMSO
AKTVIII
DMSO
AKTVIII
DMSO
AKTVIII
DMSO
AKTVIII

Figure S9

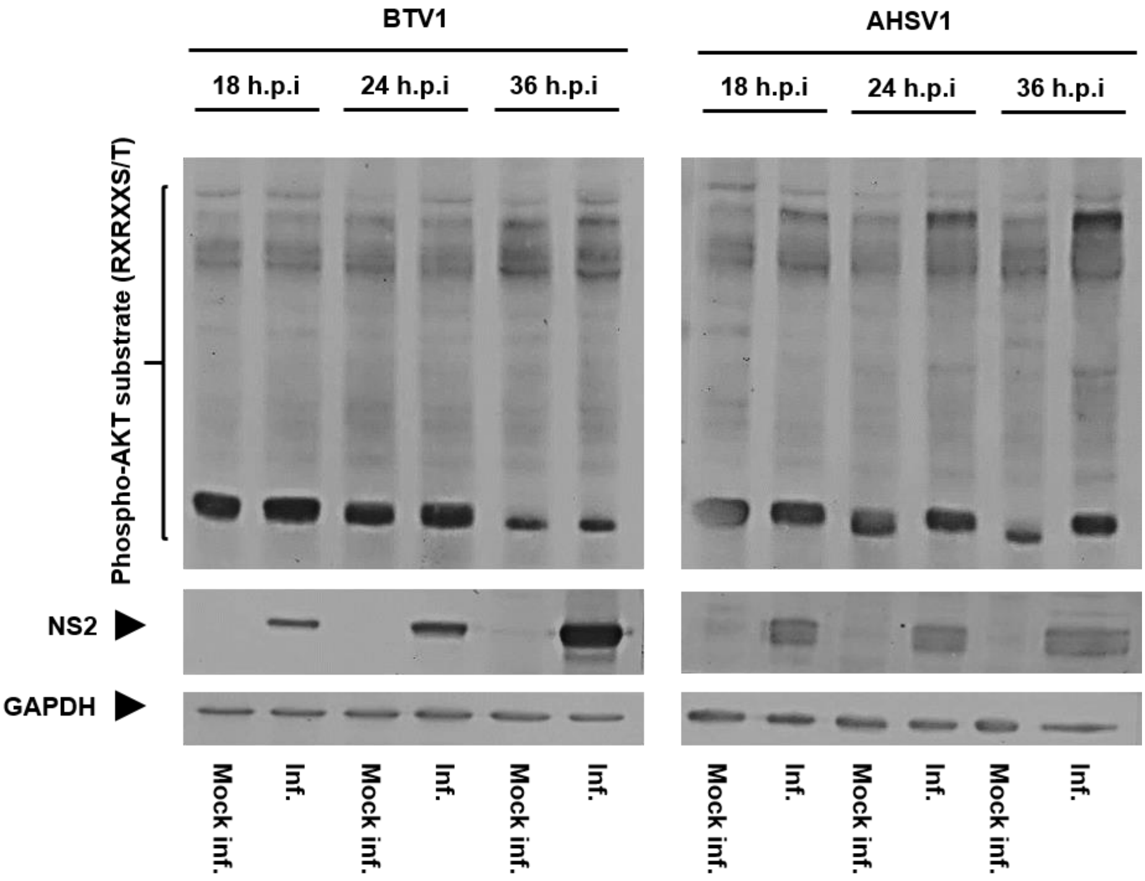
BTV1
AHSV1
18 h.p.i
24 h.p.i
36 h.p.i
Phospho-AKT substrate (RXRXXS/T)
NS2
GAPDH
Mock inf.
Inf.

Figure S10

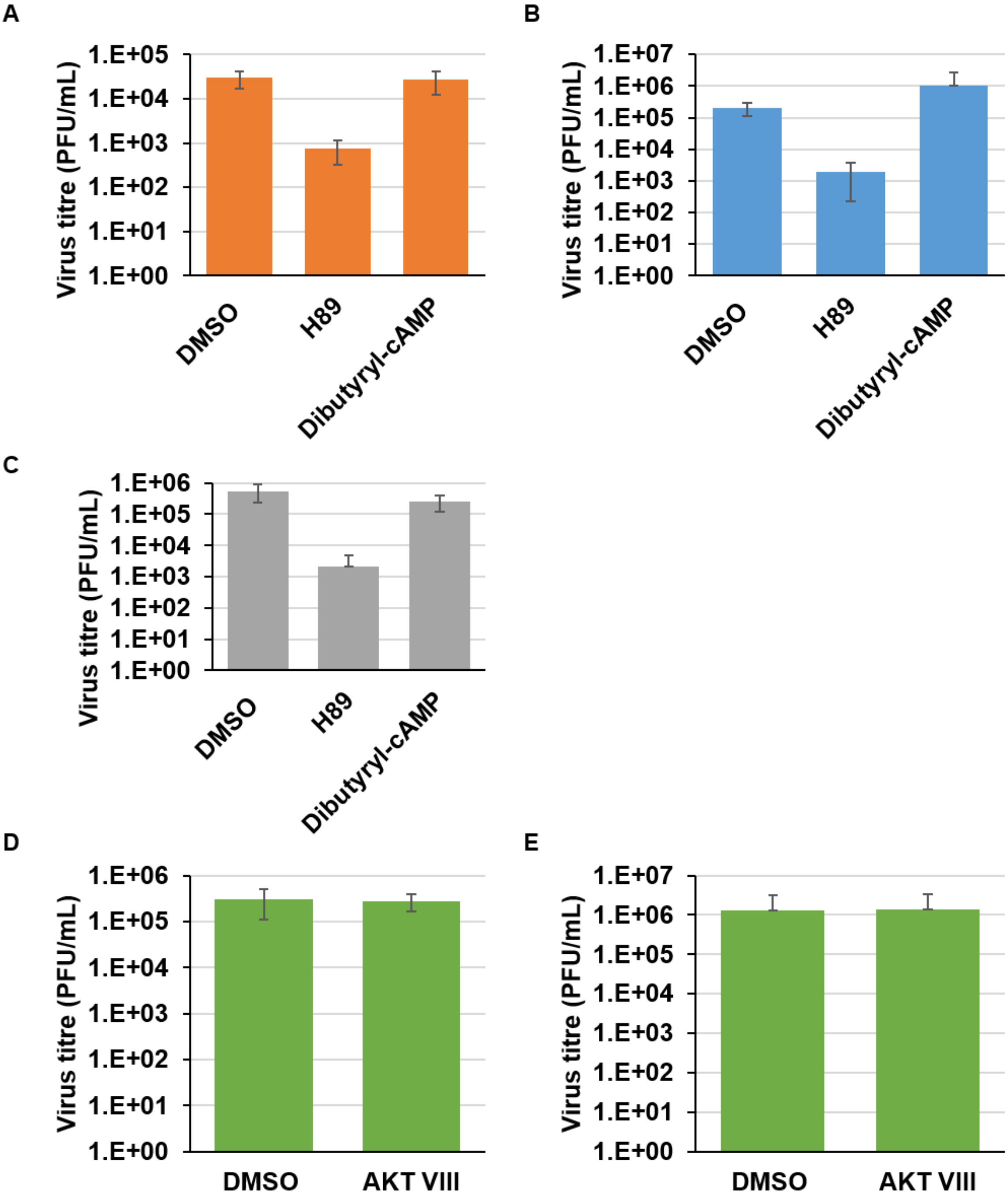
A
Virus titre (PFU/mL)
1.E+05
1.E+04
1.E+03
1.E+02
1.E+01
1.E+00
DMSO
H89
Dibutyryl-cAMP
B
Virus titre (PFU/mL)
1.E+07
1.E+06
1.E+05
1.E+04
1.E+03
1.E+02
1.E+01
1.E+00
DMSO
H89
Dibutyryl-cAMP
C
Virus titre (PFU/mL)
1.E+06
1.E+05
1.E+04
1.E+03
1.E+02
1.E+01
1.E+00
DMSO
H89
Dibutyryl-cAMP
D
Virus titre (PFU/mL)
1.E+06
1.E+05
1.E+04
1.E+03
1.E+02
1.E+01
1.E+00
DMSO
AKT VIII
E
Virus titre (PFU/mL)
1.E+07
1.E+06
1.E+05
1.E+04
1.E+03
1.E+02
1.E+01
1.E+00
DMSO
AKT VIII
