# Supplementary material for: Phosphoproteomic Analysis Reveals the Importance of Kinase Regulation During Orbivirus Infection
Source: Mol Cell Proteomics. 2017 Aug 29;16(11):1990–2005. doi: 10.1074/mcp.M117.067355 (PMC5672004; doi:10.1074/mcp.M117.067355)
Supplement: Supplemental Data [file 10.1074_M117.067355_mcp.M117.067355-2.pdf]

Supplementary Figure legends:

Figure S1. Summary data from phosphoproteomic analysis of BTV-infected HeLa cells. A) Enrichment levels of phosphopeptides within the dataset. B) Numbers of phosphopeptides identified following either Fe-NTA or TiO_2 enrichment. The percentage of phosphorylated residues within each phosphopeptide following C) Fe-NTA or D) TiO_2 enrichment are shown.

Figure S2. Correlation of the three biological replicates of the BTV phosphoproteome. Multi-scatter plots showing the correlation between the various experimental samples at A) 12h post-infection and B) 18h post-infection are given. The numbers in blue represent Pearsons correlation coefficient. The second experiment had the medium and heavy SILAC media switched to control for the impact of SILAC labeling on the results.

Figure S3. Variation in phosphorylation status upon BTV infection. Scatter plots show the normalized log2 fold change upon BTV infection against log10 p-value. Phosphosites which were significantly altered in abundance by t-test ($p<0.05$) are highlighted in green. The scatter plots represent phosphosites at A) 12h post-infection, B) 18h post-infection and C) 18h post-infection compared with 12h post-infection.

Figure S4. Identification of sequence motifs regulated by BTV infection. Phosphosites showing a significant (t-test, $p<0.05$) increase or decrease in the abundance were submitted to the weblogo software for preparation of sequence logos. Data from A) 12h post-infection, B) 18h post-infection and C) 18h compared to 12h post-infection with BTV is shown.

Figure S5. Inhibition of PKA reduces BTV replication while further stimulation of PKA enhances BTV replication. HeLa and sheep PT cells infected with BTV1 (MOI=5) were treated 1 h.p.i with DMSO, 40 μM H89 or 1 mM Dibutyryl-cAMP and harvested 12 h.p.i (A) and 18 h.p.i (B). Samples were analyzed by western blot with antibodies against the indicated antigens. Brackets at the side of the phosphorylated PKA substrate western blot denote region used for densitometry analysis.

Figure S6. Decreases or increases in PKA-dependent phosphorylated substrate levels prior to infection do not further impair or enhance BTV replication. HeLa and sheep PT cells were treated with DMSO, 40 μM H89 or 1 mM Dibutyryl-cAMP for 1 hour prior to infection with BTV1 (MOI=5) and harvested 18 h.p.i. Samples were analyzed by western blot with the indicated antibodies. Brackets at the side of the phosphorylated PKA substrate western blot denote region used for densitometry analysis.

Figure S7. Decreases or increases in PKA-dependent phosphorylated substrates post or prior to infection do not further impair or enhance AHSV replication. . HeLa and Equine dermal (E. Derm) cells were treated 1 h.p.i (A) or for 1 hour prior to infection (B) with AHSV1 (MOI=5) with DMSO, 40 μM H89 or 1

mM Dibutyryl-cAMP and harvested 18 h.p.i. Samples were analyzed by western blot with the indicated antibodies. Brackets at the side of the phosphorylated PKA substrate western blot denote region used for densitometry analysis.

Figure S8. BTV1 and AHSV1 increase AKT-dependent phosphorylated substrates. HeLa and sheep PT cells infected with BTV1 (MOI=5) (A) or HeLa and Equine dermal (E. Derm) cells infected with AHSV1 (MOI=5) (B) were treated 1 h.p.i with 4 μM Akt Inhibitor VIII (AKT VIII) and harvested 18 h.p.i. Samples were analyzed by western blot with the indicated antibodies. Brackets at the side of the phosphorylated AKT substrate western blot denote region used for densitometry analysis.

Figure S9. AKT-dependent phosphorylated substrates decrease in BTV1 infected cells but remain elevated in AHSV1 infected cells between 18 h.p.i and 36 h.p.i. HeLa cells infected with BTV1 (MOI=5) (A) or HeLa cells infected with AHSV1 (MOI=5) (B) where harvested 18 h.p.i, 24 h.p.i and 36 h.p.i. Samples were analyzed by western blot with the indicated antibodies. Brackets at the side of the phosphorylated AKT substrate western blot denote region used for densitometry analysis.

Figure S10. Inhibition of PKA, but not AKT, decreases BTV1 and AHSV1 virus titres. BSR cells were infected with virus recovered from previously infected HeLa cells (in the presence or absence of PKA activator or inhibitors and AKT inhibitor). Virus titres (Pfu/mL) were determined for BTV1 12 h.p.i (A) and 18 h.p.i. (B), and for AHSV1 18 h.p.i. (C) following treatment with 40 μM H89 or 1 mM Dibutyryl-cAMP. Virus titres (Pfu/mL) were determined for BTV1 (D) and AHSV1 (E) 18 h.p.i following treatment with 4 μM Akt Inhibitor VIII (AKT VIII). Error bars represent the SD values from three independent experiments.

Table S1. Phosphoproteins identified from BTV-infected HeLa cells. Phosphoproteins identified from BTV-infected HeLa cells. A phosphoprotein was considered to be regulated by BTV infection if at least one of its corresponding phosphosites showed regulation. Only high confidence phosphosites identified in at least 2 replicates, with a localization score of ≥0.75 were used for this analysis. Phosphoproteins identified as regulated overall, or at a given timepoint are highlighted (t-test, $p<0.05$).

Table S2. Phosphopeptides identified from BTV-infected HeLa cells. This table represents all the phosphopeptides identified within this experiment following removal of contaminants and reverse database hits. The first tab holds redundant information on all the phosphopeptides identified within each experiment and enrichment condition. The second tab holds non-redundant data on all the high confidence phosphopeptides. Phosphopeptides identified as regulated by BTV infection overall, or at a given time point are highlighted (t-test, $p<0.05$). Visualisation of all spectra is possible by download of PRIDE dataset PXD005550 and visualization in Maxquant version 1.5.7.4. For ease of viewing several columns are by default hidden which can be examined to reveal data on the ratio variability, and t-test difference and p-values for each peptide.

Table S3. Phosphosites identified from BTV-infected HeLa cells. High confidence phosphosites, possessing a localization probability of >0.75, and identified in at least two of three replicates are presented. Phosphosites identified as regulated by BTV infection overall, or at a given time point are highlighted (t-test, $p<0.05$).

Table S4. Differentially regulated phosphoproteins, phosphopeptides and phosphosites identified from BTV-infected HeLa cells. Phosphoproteins, phosphopeptides and phosphosites identified as differentially regulated by BTV infection are shown on separate tabs. A phosphoprotein was considered to be regulated if at least one phosphosite corresponding to this protein showed regulation. The relevant regulated phosphosites are show beneath each protein on the phosphoprotein tab. High confidence phosphosites, possessing a localization probability of >0.75, and identified in at least two of three replicates are presented. Phosphosites identified as regulated by BTV infection overall, or at a given time point are highlighted. In all cases regulation by BTV was determined by t-test ($p<0.05$).

Table S5. Gene ontology analysis of differentially regulated phosphoproteins from BTV-infected HeLa cells. Analysis of differentially regulated phosphoproteins (Table S1) was performed with STRING 10.5 (https://string-db.org). A phosphoprotein was considered to be significantly regulated if at least one phosphosite corresponding to that phosphoprotein showed significantly altered abundance (t-test, $p<0.05$) Analyses corresponding to GO molecular function, GO biological process, GO cellular component and KEGG are given on separate tags.

Table S6. Motif-X analysis of phosphosites significantly regulated during BTV infection. The Motif-X software was used to identify regulation of particular phosphorylation motifs by BTV infection. Those phosphosites showing significantly altered abundance by t-test ($p<0.05$) were input into the Motif-X software to look for overrepresentation compared to the human proteome at large. Fold-increase represents the degree of this overrepresentation. Different tabs hold data representing sequence motifs over-represented at 12h, 18h and 18h compared to 12h post-infection with BTV. A futher 'Total dataset' tab shows motifs identified from the whole high confidence phosphosite dataset (table S3) without prior selection of significantly altered sites.

Table S7. Phoxtrack analysis to identify kinase regulation during BTV-infection. The Phoxtrack software was used to characterize kinase regulation at 12h, 18h and 18h compared to 12h post-infection with BTV. Both kinase regulation, and the annotation of individual phosphosites to the relevant kinase is shown. These data are presented on separate tabs within the file. On the kinase tables the columns correspond to the kinase of interest, database the kinase data was obtained from, enrichment and normalized enrichment values, p-value, false discovery rate and the Phoxtrack score which combines the directionality of the NEV with the FDR. One the phosphosite tabs, the experimental value represents the log2 fold-change observed with this phosphosite, and core-enrichment refers

to whether a individual phosphosite was a primary contributor to a individual kinase score.
